# Supplementary material for: Plastid Phylogenomics and Plastomic Diversity of the Extant Lycophytes
Source: Genes (Basel). 2022 Jul 19;13(7):1280. doi: 10.3390/genes13071280 (PMC9316050; doi:10.3390/genes13071280)
Supplement: Supplementary file 1 [file genes-13-01280-s001.zip › Supplementary_Material.pdf]

# Supplementary Material

## Supplementary Figures and Tables

**Supplementary Table S1.** Sample information and basic characteristics of 81 Lycophyte and 12 outgroup plastomes. Newly sequenced plastomes are marked out and shown in bold.

| Family | Species | Genes | Genome size (bp) | GC Content (%) | IR size (bp) | IR GC (%) | SC size (bp) | SC GC (%) | GenBank ID |
|---|---|---|---|---|---|---|---|---|---|
| Isoetaceae | *Isoetes aff. amazonica* | 120 | 142,880 | 37.8 | 12,816 | 47.9 | 117,248 | 38.7 | MW255546 |
| Isoetaceae | *Isoetes aff. luetzelburgii* | 123 | 143,252 | 37.8 | 12,814 | 47.9 | 117,624 | 51.7 | MW255541 |
| Isoetaceae | *Isoetes aff. luetzelburgii* | 120 | 143,346 | 37.7 | 12,815 | 47.9 | 117,716 | 31.3 | MW255544 |
| Isoetaceae | *Isoetes aff. luetzelburgii* | 120 | 143,364 | 37.8 | 12,822 | 47.9 | 117,720 | 51.7 | MW255542 |
| Isoetaceae | *Isoetes aff. panamensis* | 120 | 143,308 | 37.7 | 12,815 | 47.9 | 117,678 | 51.7 | MW255543 |
| Isoetaceae | *Isoetes aff. panamensis* | 120 | 143,330 | 37.7 | 12,745 | 48.0 | 117,840 | 42.5 | MW255545 |
| Isoetaceae | *Isoetes amazonica* | 120 | 143,339 | 37.7 | 12,817 | 47.9 | 117,705 | 43.0 | MW255547 |
| Isoetaceae | *Isoetes butleri* | 136 | 144,912 | 38.0 | 13,099 | 48.0 | 118,714 | 35.7 | MG668891 |
| Isoetaceae | *Isoetes cangae* | 119 | 143,380 | 37.7 | 12,815 | 47.9 | 117,750 | 42.4 | MG019393 |
| Isoetaceae | *Isoetes cangae* | 119 | 143,412 | 37.7 | 12,815 | 47.9 | 117,782 | 42.4 | MG019394 |
| Isoetaceae | *Isoetes engelmannii* | 136 | 144,817 | 38.0 | 13,056 | 48.0 | 118,705 | 40.9 | MG668892 |
| Isoetaceae | *Isoetes flaccida* | 136 | 145,303 | 37.9 | 13,118 | 48.0 | 119,067 | 40.6 | GU191333 |
| Isoetaceae | *Isoetes flaccida var. chapmanii* | 136 | 145,096 | 38.0 | 13,099 | 48.0 | 118,898 | 40.9 | MG599108 |
| Isoetaceae | *Isoetes flaccida var. flaccida* | 136 | 145,126 | 38.0 | 13,116 | 48.0 | 118,894 | 46.3 | MG668893 |

| | | | | | | | | | |
|---|---|---|---|---|---|---|---|---|---|
| lsoetaceae | *Isoetes gardneriana* | 120 | 143,326 | 37.7 | 12,815 | 47.9 | 117,696 | 29.2 | MW255548 |
| lsoetaceae | *Isoetes graniticola* | 136 | 145,118 | 38.0 | 13,094 | 48.0 | 118,930 | 40.9 | MG792132 |
| lsoetaceae | *Isoetes hypsophila* | 134 | 146,362 | 38.1 | 13,691 | 47.8 | 118,980 | 32.1 | MW405450 |
| lsoetaceae | *Isoetes japonica* | 134 | 145,517 | 38.0 | 13,204 | 47.9 | 119,109 | 37.3 | MZ596344 |
| lsoetaceae | *Isoetes malinverniana* | 130 | 145,535 | 38.0 | 13,217 | 47.9 | 119,101 | 45.5 | MH549640 |
| lsoetaceae | *Isoetes mattaponica* | 136 | 145,065 | 38.0 | 13,078 | 48.0 | 118,909 | 33.3 | MG792141 |
| lsoetaceae | *Isoetes melanopoda subsp. melanopoda* | 136 | 145,075 | 38.0 | 13,099 | 48.0 | 118,877 | 40.9 | MG668895 |
| lsoetaceae | *Isoetes melanopoda subsp. silvatica* | 136 | 145,109 | 38.0 | 13,078 | 48.0 | 118,953 | 35.7 | MG668896 |
| lsoetaceae | *Isoetes melanospora* | 136 | 145,045 | 38.0 | 13,093 | 48.0 | 118,859 | 35.7 | MG668897 |
| lsoetaceae | *Isoetes nuttallii* | 136 | 144,680 | 38.2 | 13,319 | 48.0 | 118,042 | 42.3 | MG668899 |
| lsoetaceae | *Isoetes panamensis* | 120 | 143,331 | 37.7 | 12,815 | 47.9 | 117,701 | 34.2 | MW255549 |
| lsoetaceae | *Isoetes piedmontana* | 136 | 145,030 | 38.0 | 13,042 | 48.0 | 118,946 | 40.9 | MH549641 |
| lsoetaceae | *Isoetes serracarajensis* | 119 | 143,380 | 37.7 | 12,815 | 47.9 | 117,750 | 36.9 | MG019395 |
| lsoetaceae | *Isoetes sinensis* | 135 | 145,490 | 38.0 | 13,207 | 48.0 | 119,076 | 37.3 | MN172503 |
| lsoetaceae | *Isoetes triangula* | 120 | 143,357 | 37.7 | 12,816 | 47.9 | 117,725 | 33.9 | MW255550 |
| lsoetaceae | *Isoetes valida* | 136 | 145,132 | 38.0 | 13,104 | 48.0 | 118,924 | 40.9 | MG668902 |
| lsoetaceae | *Isoetes yunguiensis* | 135 | 145,355 | 38.0 | 13,065 | 48.0 | 119,225 | 34.7 | MK047605 |
| **(newly sequenced)** Lycopodiaceae | *Dendrolycopodium obscurum* | 131 | 160,200 | 35.0 | 17,421 | 42.5 | 125,358 | 32.1 | **ON357647** |

| | | | | | | | | | |
|---|---|---|---|---|---|---|---|---|---|
| Lycopodiaceae | *Dendrolycopodium obscurum* | 133 | 160,877 | 35.0 | 17,742 | 42.3 | 125,393 | 32.1 | MH549637 |
| **(newly sequenced)** Lycopodiaceae | *Dendrolycopodium verticale* | 131 | 159,990 | 35.0 | 17,349 | 42.6 | 125,292 | 31.7 | **ON357646** |
| **(newly sequenced)** Lycopodiaceae | *Diphasiastrum complanatum* | 131 | 158,543 | 35.8 | 16,370 | 43.9 | 125,803 | 34.6 | **ON357644** |
| Lycopodiaceae | *Diphasiastrum digitatum* | 133 | 159,614 | 35.7 | 16,885 | 43.4 | 125,844 | 34.1 | MH549638 |
| **(newly sequenced)** Lycopodiaceae | *Lycopodiastrum casuarinoides* | 131 | 166,814 | 32.9 | 22,272 | 40.1 | 122,270 | 34.2 | **ON357642** |
| Lycopodiaceae | *Lycopodium clavatum* | 133 | 151,819 | 34.5 | 12,417 | 46.6 | 126,985 | 36.6 | MH549642 |
| **(newly sequenced)** Lycopodiaceae | *Lycopodium japonicum* | 131 | 157,518 | 34.3 | 15,255 | 43.5 | 127,008 | 33.4 | **ON357645** |
| **(newly sequenced)** Lycopodiaceae | *Palhinhaea cernua* | 129 | 145,297 | 35.4 | 12,801 | 46.7 | 119,695 | 27.5 | **ON357648** |
| **(newly sequenced)** Lycopodiaceae | *Palhinhaea cernua* | 129 | 145,398 | 35.5 | 12,288 | 47.0 | 120,822 | 30.2 | **ON357650** |
| **(newly sequenced)** Lycopodiaceae | *Palhinhaea cernua* | 129 | 145,564 | 35.2 | 12,999 | 46.2 | 119,566 | 28.9 | **ON357649** |
| **(newly sequenced)** Lycopodiaceae | *Pseudolycopodiella caroliniana* | 130 | 147,356 | 34.1 | 13,850 | 45.6 | 119,656 | 30.9 | **ON357643** |
| Lycopodiaceae | *Phlegmariurus carinatus* | 130 | 150,349 | 34.0 | 15,156 | 44.2 | 120,037 | 30.1 | MN566837 |
| **(newly sequenced)** Lycopodiaceae | *Phlegmariurus henryi* | 131 | 150,450 | 33.9 | 15,293 | 44.0 | 119,864 | 30.9 | **ON357651** |

| | | | | | | | | | |
|---|---|---|---|---|---|---|---|---|---|
| **(newly sequenced)** Lycopodiaceae | *Phlegmariurus mingcheensis* | 131 | 149,043 | 34.0 | 14,959 | 44.1 | 119,125 | 30.2 | **ON357652** |
| Lycopodiaceae | *Phlegmariurus phlegmaria* | 130 | 149,711 | 33.8 | 15,192 | 44.0 | 119,327 | 35.1 | MT786212 |
| **(newly sequenced)** Lycopodiaceae | *Phlegmariurus squarrosus* | 131 | 150,256 | 34.0 | 15,155 | 44.2 | 119,946 | 31.2 | **ON357653** |
| **(newly sequenced)** Lycopodiaceae | *Huperzia crispata* | 131 | 153,825 | 36.3 | 15,144 | 45.0 | 123,537 | 41.4 | **ON400074** |
| Lycopodiaceae | *Huperzia javanica* | 129 | 154,415 | 36.4 | 15,314 | 45.0 | 123,787 | 40.4 | KY609860 |
| Lycopodiaceae | *Huperzia lucidula* | 132 | 154,368 | 36.3 | 15,314 | 44.9 | 123,740 | 40.9 | MH549639 |
| Lycopodiaceae | *Huperzia lucidula* | 131 | 154,373 | 36.2 | 15,314 | 44.9 | 123,745 | 40.8 | AY660566 |
| **(newly sequenced)** Lycopodiaceae | *Huperzia selago* | 131 | 168,075 | 36.0 | 22,261 | 41.3 | 123,553 | 36.3 | **ON357641** |
| Lycopodiaceae | *Huperzia serrata* | 131 | 154,176 | 36.3 | 15,313 | 44.9 | 123,550 | 41.2 | KX426071 |
| Selaginellaceae | *Selaginella bisulcata* | 104 | 140,509 | 52.8 | 12,626 | 54.2 | 115,257 | 47.4 | MH598531 |
| Selaginellaceae | *Selaginella doederleinii* | 103 | 142,752 | 51.1 | 11,023 | 55.8 | 120,706 | 47.5 | MH598532 |
| Selaginellaceae | *Selaginella exaltata* | 80 | 117,523 | 51.8 | 12,624 | 55.9 | 92,275 | 32.5 | MN427927 |
| Selaginellaceae | *Selaginella hainanensis* | 107 | 144,201 | 54.8 | 12,801 | 57.4 | 118,599 | 55.3 | MH598533 |
| Selaginellaceae | *Selaginella indica* | 87 | 122,460 | 53.6 | 14,177 | 56.2 | 94,106 | 52.8 | MK156801 |
| Selaginellaceae | *Selaginella involvens* | 102 | 143,192 | 50.8 | 11,962 | 55.8 | 119,268 | 52.0 | MK460599 |
| Selaginellaceae | *Selaginella kraussiana* | 94 | 129,971 | 52.3 | 14,597 | 56.5 | 100,777 | 48.9 | MH549643 |
| Selaginellaceae | *Selaginella lepidophylla* | 87 | 114,693 | 51.9 | 7,308 | 57.1 | 100,077 | 47.1 | MK089531 |

| | | | | | | | | | |
|---|---|---|---|---|---|---|---|---|---|
| Selaginellaceae | *Selaginella lyallii* | 84 | 110,411 | 50.7 | 10,096 | 55.9 | 90,219 | 47.5 | MK156800 |
| Selaginellaceae | *Selaginella moellendorffii* | 101 | 143,525 | 51.0 | 12,099 | 55.8 | 119,327 | 54.4 | MG272484 |
| Selaginellaceae | *Selaginella moellendorffii* | 106 | 143,775 | 51.0 | 12,114 | 55.7 | 119,547 | 50.8 | HM173080 |
| Selaginellaceae | *Selaginella moellendorffii* | 100 | 143,780 | 51.0 | 12,090 | 55.7 | 119,600 | 54.4 | FJ755183 |
| Selaginellaceae | *Selaginella nipponica* | 65 | 110,164 | 53.5 | 18,967 | 55.1 | 72,230 | 58.7 | MK293726 |
| Selaginellaceae | *Selaginella nummularifolia* | 110 | 148,924 | 50.2 | 18,511 | 52.1 | 111,902 | 36.1 | MK622381 |
| Selaginellaceae | *Selaginella pallidissima* | 64 | 110,227 | 53.5 | 18,957 | 55.1 | 72,313 | 58.6 | MK293728 |
| Selaginellaceae | *Selaginella pennata* | 99 | 138,024 | 52.9 | 11,599 | 54.6 | 114,826 | 50.9 | MH598534 |
| Selaginellaceae | *Selaginella remotifolia* | 96 | 131,867 | 56.5 | 14,836 | 57.7 | 102,195 | 45.7 | MH598535 |
| Selaginellaceae | *Selaginella rossii* | 111 | 146,469 | 50.7 | 17,862 | 52.0 | 110,745 | 33.2 | MK622382 |
| Selaginellaceae | *Selaginella sanguinolenta* | 113 | 147,148 | 50.8 | 16,531 | 52.2 | 114,086 | 39.1 | MH598536 |
| Selaginellaceae | *Selaginella sanguinolenta* | 108 | 148,236 | 50.9 | 18,382 | 52.2 | 111,472 | 52.3 | MK622383 |
| Selaginellaceae | *Selaginella stauntoniana* | 80 | 126,762 | 54.1 | 12,393 | 55.2 | 101,976 | 68.1 | MK460598 |
| Selaginellaceae | *Selaginella stauntoniana* | 79 | 126,835 | 54.1 | 12,824 | 55.2 | 101,187 | 68.1 | MK622384 |
| Selaginellaceae | *Selaginella tamariscina* | 80 | 126,365 | 54.0 | 12,796 | 55.3 | 100,773 | 55.0 | MN894555 |
| Selaginellaceae | *Selaginella tamariscina* | 80 | 126,700 | 54.1 | 12,830 | 55.2 | 101,040 | 53.8 | MH598537 |
| Selaginellaceae | *Selaginella uncinata* | 107 | 144,161 | 54.9 | 12,779 | 57.5 | 118,603 | 55.0 | MG272483 |
| Selaginellaceae | *Selaginella uncinata* | 105 | 144,170 | 54.8 | 12,789 | 57.4 | 118,592 | 54.6 | AB197035 |
| Selaginellaceae | *Selaginella vardei* | 85 | 121,254 | 53.2 | 13,893 | 55.7 | 93,468 | 52.5 | MG272482 |
| (outgroup) Funariaceae | *Physcomitrium patens* | 132 | 122,890 | 28.5 | - | - | - | - | NC_005087 |

| | | | | | | | | | |
|---|---|---|---|---|---|---|---|---|---|
| (outgroup) Marchantiaceae | *Marchantia polymorpha* | 129 | 120,314 | 29.0 | - | - | - | - | NC_042505 |
| (outgroup) Anthocerotaceae | *Anthoceros punctatus* | 137 | 160,692 | 32.3 | - | - | - | - | NC_049001 |
| (outgroup) Fabaceae | *Glycine max* | 128 | 152,218 | 35.4 | - | - | - | - | NC_007942 |
| (outgroup) Marattiaceae | *Angiopteris evecta* | 141 | 153,901 | 35.5 | - | - | - | - | NC_008829 |
| (outgroup) Brassicaceae | *Arabidopsis thaliana* | 129 | 154,478 | 36.3 | - | - | - | - | NC_000932 |
| (outgroup) Pteridaceae | *Pteris vittata* | 128 | 149,424 | 36.7 | - | - | - | - | NC_040204 |
| (outgroup) Pinaceae | *Abies fabri* | 113 | 120,027 | 38.3 | - | - | - | - | NC_057314 |
| (outgroup) Nymphaeaceae | *Nymphaea tetragona* | 128 | 159,956 | 39.1 | - | - | - | - | NC_057565 |
| (outgroup) Cycadaceae | *Cycas revoluta* | 156 | 162,489 | 39.4 | - | - | - | - | NC_020319 |
| (outgroup) Ginkgoaceae | *Ginkgo biloba* | 131 | 156,988 | 39.6 | - | - | - | - | NC_016986 |
| (outgroup) Cyatheaceae | *Alsophila spinulosa* | 133 | 156,661 | 40.4 | - | - | - | - | NC_012818 |

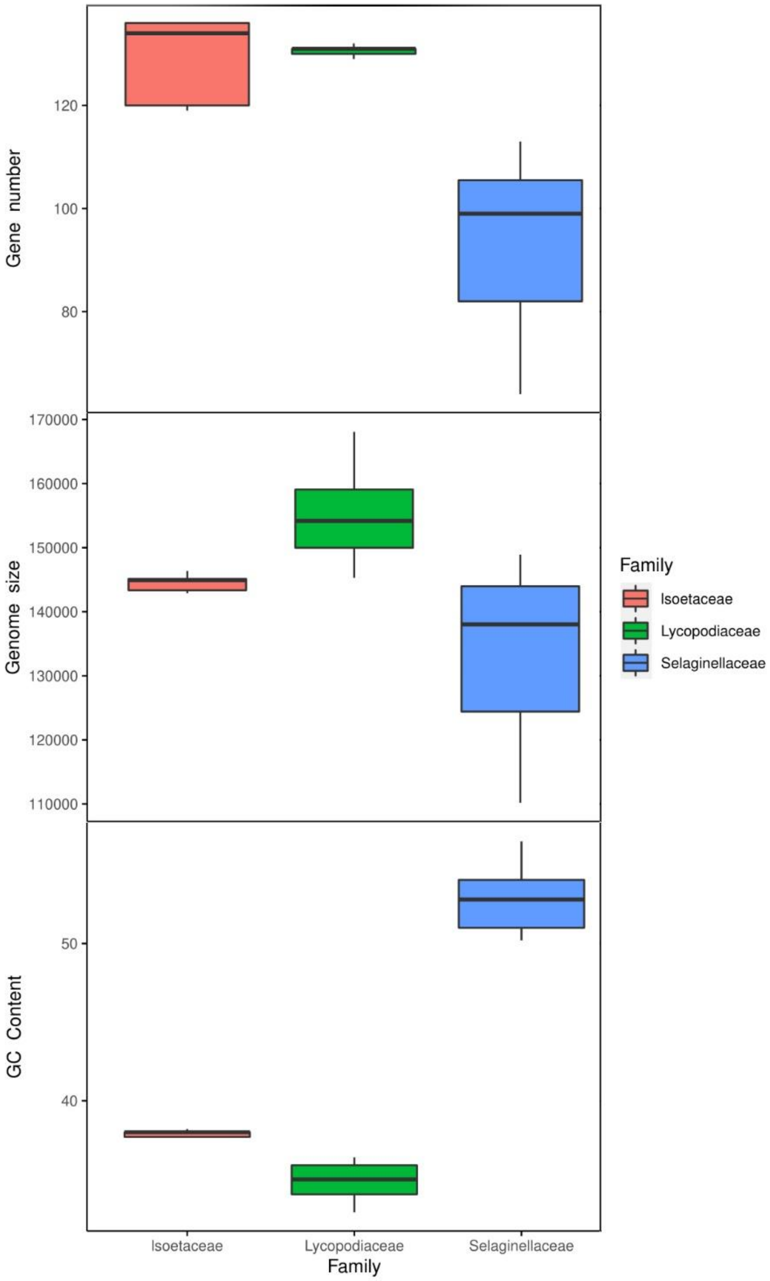


**Supplementary Figure S1.** Boxplot showing variation of GC content, genome size and gene number of three families of the lycophyte.

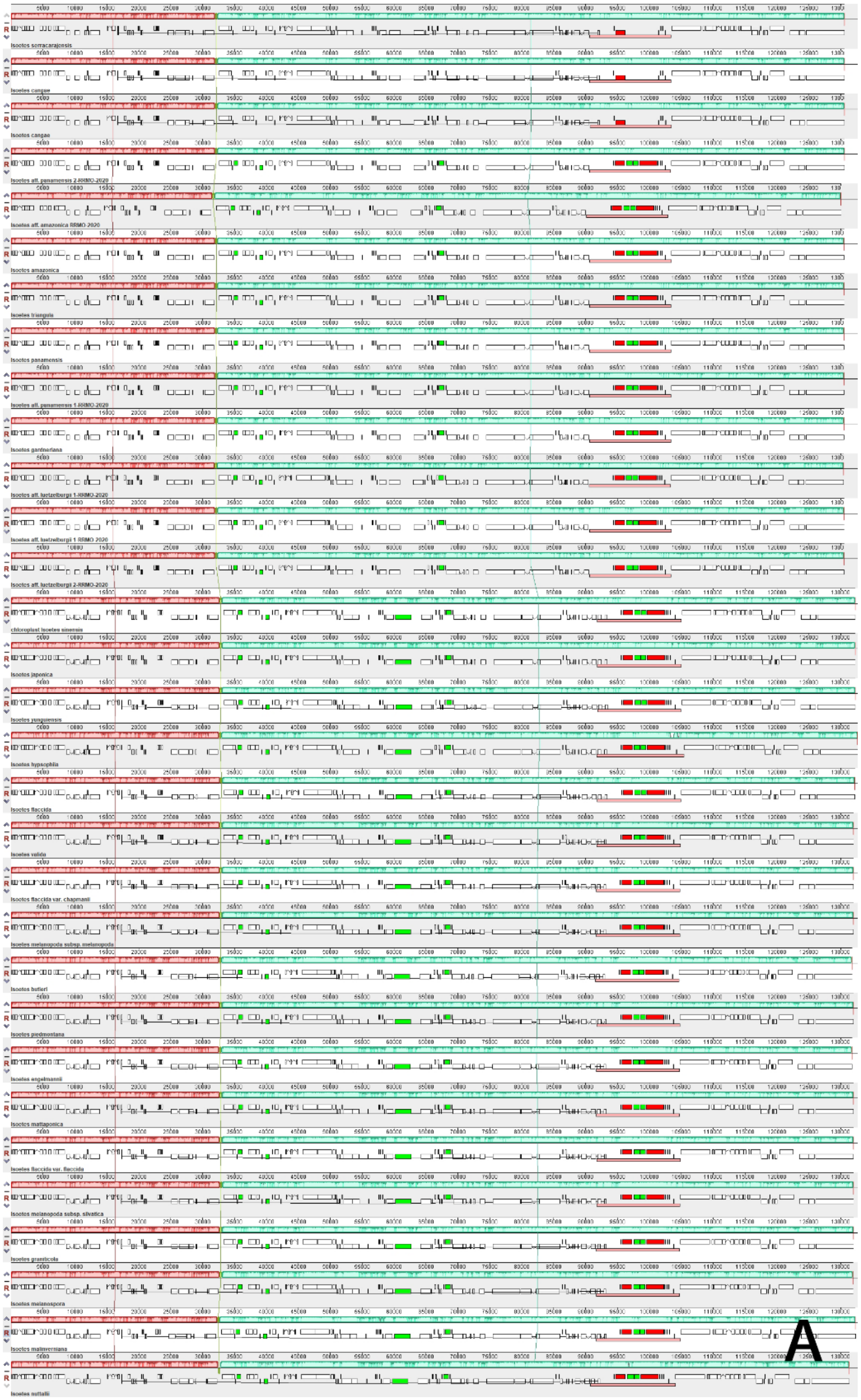
Isoetes serracarajensis
Isoetes cangae
Isoetes cangae
Isoetes aff. panamensis 2-RRMO-2020
Isoetes aff. amazonica RRMO-2020
Isoetes amazonica
Isoetes triangula
Isoetes panamensis
Isoetes aff. panamensis 1-RRMO-2020
Isoetes gardneriana
Isoetes aff. luetzelburgii 1-RRMO-2020
Isoetes aff. luetzelburgii 1-RRMO-2020
Isoetes aff. luetzelburgii 2-RRMO-2020
chloroplast Isoetes sinensis
Isoetes japonica
Isoetes yunguiensis
Isoetes hypsophila
Isoetes flaccida
Isoetes valida
Isoetes flaccida var. chapmanii
Isoetes melanopoda subsp. melanopoda
Isoetes butleri
Isoetes piedmontana
Isoetes engelmannii
Isoetes mattaponica
Isoetes flaccida var. flaccida
Isoetes melanopoda subsp. silvatica
Isoetes graniticola
Isoetes melanospora
Isoetes malinverniana
Isoetes nuttallii
A

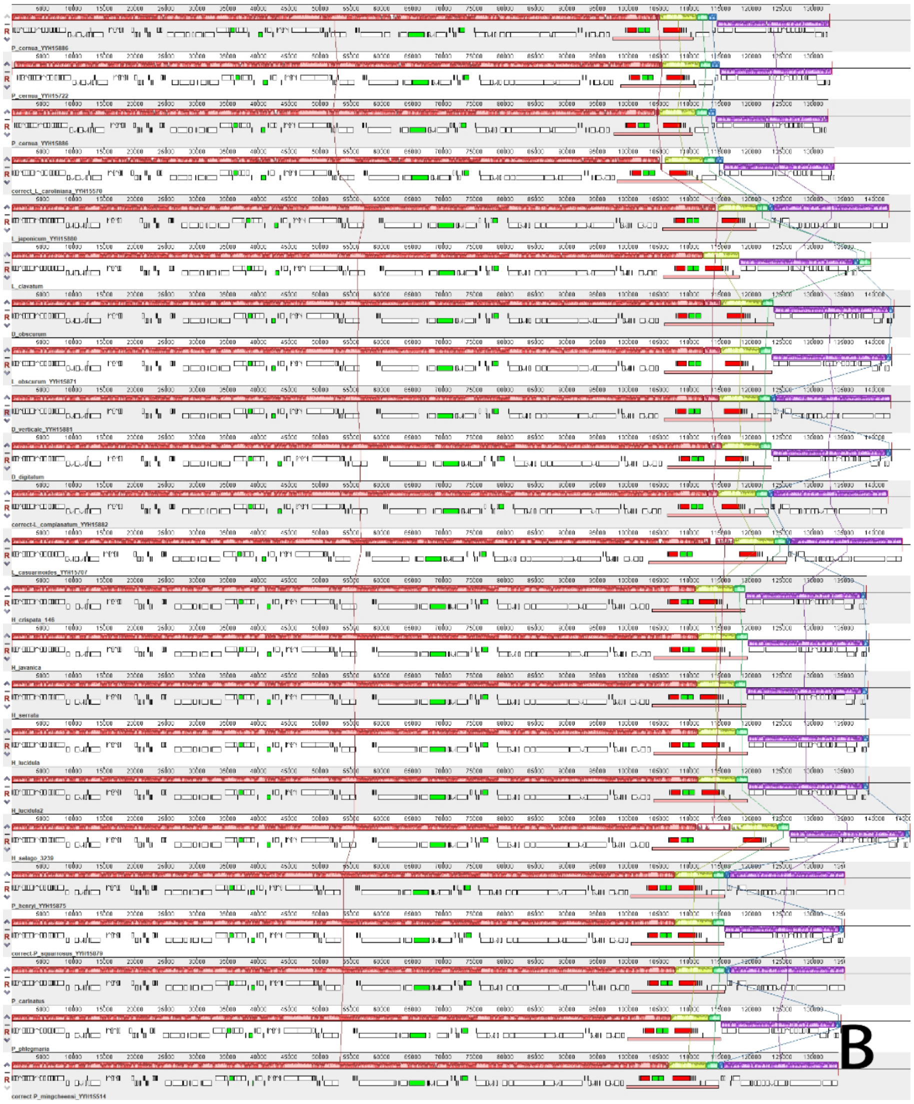
B

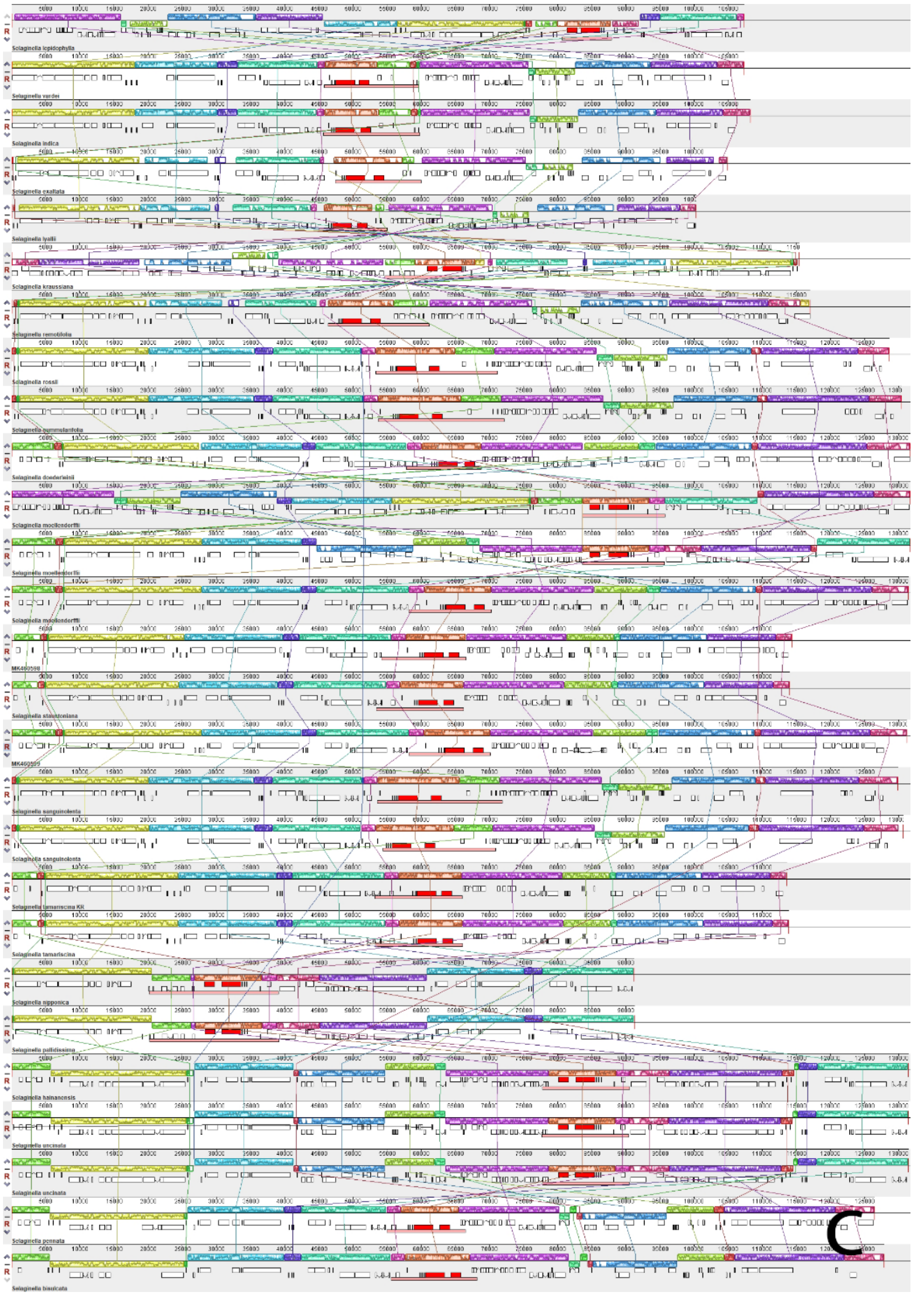

**Supplementary Figure S2.** MAUVE alignment of the plastomes of three families. A. Isoetaceae; B. Lycopodiaceae and C. Selaginellaceae. The last repeat region is removed before the alignment. Within each of the alignments, locally collinear blocks (LCBs) are represented as boxes of the same color connected by lines, with the average level of sequence conservation indicated by the height of the block profiles.

**Supplementary Files S2.** Best partition schemes of PartitionFinder2 used in the phylogenetic analyses (IQtree and MrBayes).

# DATA BLOCKS #
[data_blocks]
ycf1_mfa_gb_codon1=1-3240\3;

ycf1_mfa_gb_codon2=2-3240\3;
ycf1_mfa_gb_codon3=3-3240\3;
rpoC1_mfa_gb_codon1=3241-5160\3;
rpoC1_mfa_gb_codon2=3242-5160\3;
rpoC1_mfa_gb_codon3=3243-5160\3;
rps2_mfa_gb_codon1=5161-5838\3;
rps2_mfa_gb_codon2=5162-5838\3;
rps2_mfa_gb_codon3=5163-5838\3;
ycf2_mfa_gb_codon1=5839-9099\3;
ycf2_mfa_gb_codon2=5840-9099\3;
ycf2_mfa_gb_codon3=5841-9099\3;
rpl2_mfa_gb_codon1=9100-9903\3;
rpl2_mfa_gb_codon2=9101-9903\3;
rpl2_mfa_gb_codon3=9102-9903\3;
rpoC2_mfa_gb_codon1=9904-13386\3;
rpoC2_mfa_gb_codon2=9905-13386\3;
rpoC2_mfa_gb_codon3=9906-13386\3;
rps3_mfa_gb_codon1=13387-14022\3;
rps3_mfa_gb_codon2=13388-14022\3;
rps3_mfa_gb_codon3=13389-14022\3;
ycf3_mfa_gb_codon1=14023-14367\3;
ycf3_mfa_gb_codon2=14024-14367\3;
ycf3_mfa_gb_codon3=14025-14367\3;
rps4_mfa_gb_codon1=14368-14913\3;
rps4_mfa_gb_codon2=14369-14913\3;
rps4_mfa_gb_codon3=14370-14913\3;
ycf4_mfa_gb_codon1=14914-15426\3;
ycf4_mfa_gb_codon2=14915-15426\3;
ycf4_mfa_gb_codon3=14916-15426\3;
rps7_mfa_gb_codon1=15427-15882\3;
rps7_mfa_gb_codon2=15428-15882\3;
rps7_mfa_gb_codon3=15429-15882\3;
rps8_mfa_gb_codon1=15883-16263\3;
rps8_mfa_gb_codon2=15884-16263\3;
rps8_mfa_gb_codon3=15885-16263\3;
rps11_mfa_gb_codon1=16264-16632\3;
rps11_mfa_gb_codon2=16265-16632\3;
rps11_mfa_gb_codon3=16266-16632\3;
rps12_mfa_gb_codon1=16633-16743\3;
rps12_mfa_gb_codon2=16634-16743\3;
rps12_mfa_gb_codon3=16635-16743\3;
ycf12_mfa_gb_codon1=16744-16842\3;
ycf12_mfa_gb_codon2=16745-16842\3;
ycf12_mfa_gb_codon3=16746-16842\3;
rps14_mfa_gb_codon1=16843-17139\3;
rps14_mfa_gb_codon2=16844-17139\3;
rps14_mfa_gb_codon3=16845-17139\3;
rpl14_mfa_gb_codon1=17140-17502\3;
rpl14_mfa_gb_codon2=17141-17502\3;
rpl14_mfa_gb_codon3=17142-17502\3;
rps15_mfa_gb_codon1=17503-17757\3;
rps15_mfa_gb_codon2=17504-17757\3;

rps15_mfa_gb_codon3=17505-17757\3;
rpl16_mfa_gb_codon1=17758-18159\3;
rpl16_mfa_gb_codon2=17759-18159\3;
rpl16_mfa_gb_codon3=17760-18159\3;
rps18_mfa_gb_codon1=18160-18375\3;
rps18_mfa_gb_codon2=18161-18375\3;
rps18_mfa_gb_codon3=18162-18375\3;
rps19_mfa_gb_codon1=18376-18654\3;
rps19_mfa_gb_codon2=18377-18654\3;
rps19_mfa_gb_codon3=18378-18654\3;
rpl20_mfa_gb_codon1=18655-18948\3;
rpl20_mfa_gb_codon2=18656-18948\3;
rpl20_mfa_gb_codon3=18657-18948\3;
rpl21_mfa_gb_codon1=18949-19257\3;
rpl21_mfa_gb_codon2=18950-19257\3;
rpl21_mfa_gb_codon3=18951-19257\3;
rpl22_mfa_gb_codon1=19258-19602\3;
rpl22_mfa_gb_codon2=19259-19602\3;
rpl22_mfa_gb_codon3=19260-19602\3;
rpl23_mfa_gb_codon1=19603-19866\3;
rpl23_mfa_gb_codon2=19604-19866\3;
rpl23_mfa_gb_codon3=19605-19866\3;
rpl32_mfa_gb_codon1=19867-20019\3;
rpl32_mfa_gb_codon2=19868-20019\3;
rpl32_mfa_gb_codon3=19869-20019\3;
rpl33_mfa_gb_codon1=20020-20181\3;
rpl33_mfa_gb_codon2=20021-20181\3;
rpl33_mfa_gb_codon3=20022-20181\3;
rpl36_mfa_gb_codon1=20182-20280\3;
rpl36_mfa_gb_codon2=20183-20280\3;
rpl36_mfa_gb_codon3=20184-20280\3;
ycf66_mfa_gb_codon1=20281-20712\3;
ycf66_mfa_gb_codon2=20282-20712\3;
ycf66_mfa_gb_codon3=20283-20712\3;
atpA_mfa_gb_codon1=20713-22227\3;
atpA_mfa_gb_codon2=20714-22227\3;
atpA_mfa_gb_codon3=20715-22227\3;
atpB_mfa_gb_codon1=22228-23682\3;
atpB_mfa_gb_codon2=22229-23682\3;
atpB_mfa_gb_codon3=22230-23682\3;
atpE_mfa_gb_codon1=23683-24069\3;
atpE_mfa_gb_codon2=23684-24069\3;
atpE_mfa_gb_codon3=23685-24069\3;
atpF_mfa_gb_codon1=24070-24591\3;
atpF_mfa_gb_codon2=24071-24591\3;
atpF_mfa_gb_codon3=24072-24591\3;
atpH_mfa_gb_codon1=24592-24834\3;
atpH_mfa_gb_codon2=24593-24834\3;
atpH_mfa_gb_codon3=24594-24834\3;
atpI_mfa_gb_codon1=24835-25572\3;
atpI_mfa_gb_codon2=24836-25572\3;

atpI_mfa_gb_codon3=24837-25572\3;
ccsA_mfa_gb_codon1=25573-26433\3;
ccsA_mfa_gb_codon2=25574-26433\3;
ccsA_mfa_gb_codon3=25575-26433\3;
cemA_mfa_gb_codon1=26434-27753\3;
cemA_mfa_gb_codon2=26435-27753\3;
cemA_mfa_gb_codon3=26436-27753\3;
chlB_mfa_gb_codon1=27754-29274\3;
chlB_mfa_gb_codon2=27755-29274\3;
chlB_mfa_gb_codon3=27756-29274\3;
chlL_mfa_gb_codon1=29275-30123\3;
chlL_mfa_gb_codon2=29276-30123\3;
chlL_mfa_gb_codon3=29277-30123\3;
chlN_mfa_gb_codon1=30124-31455\3;
chlN_mfa_gb_codon2=30125-31455\3;
chlN_mfa_gb_codon3=30126-31455\3;
clpP_mfa_gb_codon1=31456-32028\3;
clpP_mfa_gb_codon2=31457-32028\3;
clpP_mfa_gb_codon3=31458-32028\3;
infA_mfa_gb_codon1=32029-32250\3;
infA_mfa_gb_codon2=32030-32250\3;
infA_mfa_gb_codon3=32031-32250\3;
matK_mfa_gb_codon1=32251-33591\3;
matK_mfa_gb_codon2=32252-33591\3;
matK_mfa_gb_codon3=32253-33591\3;
ndhA_mfa_gb_codon1=33592-34593\3;
ndhA_mfa_gb_codon2=33593-34593\3;
ndhA_mfa_gb_codon3=33594-34593\3;
ndhB_mfa_gb_codon1=34594-36048\3;
ndhB_mfa_gb_codon2=34595-36048\3;
ndhB_mfa_gb_codon3=34596-36048\3;
ndhC_mfa_gb_codon1=36049-36402\3;
ndhC_mfa_gb_codon2=36050-36402\3;
ndhC_mfa_gb_codon3=36051-36402\3;
ndhD_mfa_gb_codon1=36403-37857\3;
ndhD_mfa_gb_codon2=36404-37857\3;
ndhD_mfa_gb_codon3=36405-37857\3;
ndhE_mfa_gb_codon1=37858-38148\3;
ndhE_mfa_gb_codon2=37859-38148\3;
ndhE_mfa_gb_codon3=37860-38148\3;
ndhF_mfa_gb_codon1=38149-40191\3;
ndhF_mfa_gb_codon2=38150-40191\3;
ndhF_mfa_gb_codon3=38151-40191\3;
ndhG_mfa_gb_codon1=40192-40704\3;
ndhG_mfa_gb_codon2=40193-40704\3;
ndhG_mfa_gb_codon3=40194-40704\3;
ndhH_mfa_gb_codon1=40705-41865\3;
ndhH_mfa_gb_codon2=40706-41865\3;
ndhH_mfa_gb_codon3=40707-41865\3;
ndhI_mfa_gb_codon1=41866-42348\3;
ndhI_mfa_gb_codon2=41867-42348\3;
ndhI_mfa_gb_codon3=41868-42348\3;

ndhJ_mfa_gb_codon1=42349-42765\3;
ndhJ_mfa_gb_codon2=42350-42765\3;
ndhJ_mfa_gb_codon3=42351-42765\3;
ndhK_mfa_gb_codon1=42766-43344\3;
ndhK_mfa_gb_codon2=42767-43344\3;
ndhK_mfa_gb_codon3=42768-43344\3;
petA_mfa_gb_codon1=43345-44283\3;
petA_mfa_gb_codon2=43346-44283\3;
petA_mfa_gb_codon3=43347-44283\3;
petB_mfa_gb_codon1=44284-44922\3;
petB_mfa_gb_codon2=44285-44922\3;
petB_mfa_gb_codon3=44286-44922\3;
petD_mfa_gb_codon1=44923-45393\3;
petD_mfa_gb_codon2=44924-45393\3;
petD_mfa_gb_codon3=44925-45393\3;
petG_mfa_gb_codon1=45394-45492\3;
petG_mfa_gb_codon2=45395-45492\3;
petG_mfa_gb_codon3=45396-45492\3;
petL_mfa_gb_codon1=45493-45576\3;
petL_mfa_gb_codon2=45494-45576\3;
petL_mfa_gb_codon3=45495-45576\3;
petN_mfa_gb_codon1=45577-45663\3;
petN_mfa_gb_codon2=45578-45663\3;
petN_mfa_gb_codon3=45579-45663\3;
psaA_mfa_gb_codon1=45664-47913\3;
psaA_mfa_gb_codon2=45665-47913\3;
psaA_mfa_gb_codon3=45666-47913\3;
psaB_mfa_gb_codon1=47914-50115\3;
psaB_mfa_gb_codon2=47915-50115\3;
psaB_mfa_gb_codon3=47916-50115\3;
psaC_mfa_gb_codon1=50116-50361\3;
psaC_mfa_gb_codon2=50117-50361\3;
psaC_mfa_gb_codon3=50118-50361\3;
psaI_mfa_gb_codon1=50362-50466\3;
psaI_mfa_gb_codon2=50363-50466\3;
psaI_mfa_gb_codon3=50364-50466\3;
psaJ_mfa_gb_codon1=50467-50586\3;
psaJ_mfa_gb_codon2=50468-50586\3;
psaJ_mfa_gb_codon3=50469-50586\3;
psaM_mfa_gb_codon1=50587-50673\3;
psaM_mfa_gb_codon2=50588-50673\3;
psaM_mfa_gb_codon3=50589-50673\3;
psbA_mfa_gb_codon1=50674-51732\3;
psbA_mfa_gb_codon2=50675-51732\3;
psbA_mfa_gb_codon3=50676-51732\3;
psbB_mfa_gb_codon1=51733-53250\3;
psbB_mfa_gb_codon2=51734-53250\3;
psbB_mfa_gb_codon3=51735-53250\3;
psbC_mfa_gb_codon1=53251-54666\3;
psbC_mfa_gb_codon2=53252-54666\3;
psbC_mfa_gb_codon3=53253-54666\3;

psbD_mfa_gb_codon1=54667-55674\3;
psbD_mfa_gb_codon2=54668-55674\3;
psbD_mfa_gb_codon3=54669-55674\3;
psbE_mfa_gb_codon1=55675-55926\3;
psbE_mfa_gb_codon2=55676-55926\3;
psbE_mfa_gb_codon3=55677-55926\3;
psbF_mfa_gb_codon1=55927-56046\3;
psbF_mfa_gb_codon2=55928-56046\3;
psbF_mfa_gb_codon3=55929-56046\3;
psbH_mfa_gb_codon1=56047-56262\3;
psbH_mfa_gb_codon2=56048-56262\3;
psbH_mfa_gb_codon3=56049-56262\3;
psbI_mfa_gb_codon1=56263-56370\3;
psbI_mfa_gb_codon2=56264-56370\3;
psbI_mfa_gb_codon3=56265-56370\3;
psbJ_mfa_gb_codon1=56371-56493\3;
psbJ_mfa_gb_codon2=56372-56493\3;
psbJ_mfa_gb_codon3=56373-56493\3;
psbK_mfa_gb_codon1=56494-56643\3;
psbK_mfa_gb_codon2=56495-56643\3;
psbK_mfa_gb_codon3=56496-56643\3;
psbL_mfa_gb_codon1=56644-56757\3;
psbL_mfa_gb_codon2=56645-56757\3;
psbL_mfa_gb_codon3=56646-56757\3;
psbM_mfa_gb_codon1=56758-56850\3;
psbM_mfa_gb_codon2=56759-56850\3;
psbM_mfa_gb_codon3=56760-56850\3;
psbN_mfa_gb_codon1=56851-56976\3;
psbN_mfa_gb_codon2=56852-56976\3;
psbN_mfa_gb_codon3=56853-56976\3;
psbT_mfa_gb_codon1=56977-57069\3;
psbT_mfa_gb_codon2=56978-57069\3;
psbT_mfa_gb_codon3=56979-57069\3;
psbZ_mfa_gb_codon1=57070-57255\3;
psbZ_mfa_gb_codon2=57071-57255\3;
psbZ_mfa_gb_codon3=57072-57255\3;
rbcL_mfa_gb_codon1=57256-58683\3;
rbcL_mfa_gb_codon2=57257-58683\3;
rbcL_mfa_gb_codon3=57258-58683\3;
rpoA_mfa_gb_codon1=58684-59346\3;
rpoA_mfa_gb_codon2=58685-59346\3;
rpoA_mfa_gb_codon3=58686-59346\3;
rpoB_mfa_gb_codon1=59347-62457\3;
rpoB_mfa_gb_codon2=59348-62457\3;
rpoB_mfa_gb_codon3=59349-62457\3;
Best partitioning scheme
Subset | Best Model | # sites | subset id | Partition names
1 | GTR+I+G | 3769 | 93b3cf83c40162db317fd959a5e69a9e | matK_mfa_gb_codon1, cemA_mfa_gb_codon2, ycf1_mfa_gb_codon1, rpoC2_mfa_gb_codon3, matK_mfa_gb_codon2, rps12_mfa_gb_codon1, rpl21_mfa_gb_codon1, rpl33_mfa_gb_codon3
2 | GTR+I+G | 1109 | 6b93474dc4c2552d2431625603ba0b45 | ycf1_mfa_gb_codon2, psaM_mfa_gb_codon3

3 | GTR+I+G | 1756 | 7a6fadd98e3680aa2381a6e94c4a95c1 | ycf1_mfa_gb_codon3, matK_mfa_gb_codon3, rps15_mfa_gb_codon3, ycf66_mfa_gb_codon3
4 | GTR+I+G | 5412 | 60fb556c367e49a4bd764969e3e87ea5 | chlB_mfa_gb_codon1, chlN_mfa_gb_codon1, ndhJ_mfa_gb_codon1, clpP_mfa_gb_codon3, ndhI_mfa_gb_codon1, ndhK_mfa_gb_codon2, rps14_mfa_gb_codon2, rps18_mfa_gb_codon1, infA_mfa_gb_codon1, rps2_mfa_gb_codon1, ndhE_mfa_gb_codon1, rpoB_mfa_gb_codon1, rpoC1_mfa_gb_codon1, rps4_mfa_gb_codon1, rpl22_mfa_gb_codon2, ycf4_mfa_gb_codon1, rpl36_mfa_gb_codon1, atpE_mfa_gb_codon1, atpF_mfa_gb_codon1, rps3_mfa_gb_codon1, rps8_mfa_gb_codon1, rpl14_mfa_gb_codon1, rpl2_mfa_gb_codon1
5 | GTR+I+G | 3601 | 8b49ca56a6c9d1b278c18b6a1c6b5eb3 | petL_mfa_gb_codon1, ndhB_mfa_gb_codon2, ycf12_mfa_gb_codon1, ndhA_mfa_gb_codon1, ndhC_mfa_gb_codon2, rps18_mfa_gb_codon2, ycf4_mfa_gb_codon2, psbH_mfa_gb_codon1, psbK_mfa_gb_codon1, ndhI_mfa_gb_codon2, rpoC1_mfa_gb_codon2, rpoB_mfa_gb_codon2, atpF_mfa_gb_codon2, rps2_mfa_gb_codon2
6 | GTR+I+G | 5460 | 3e36598d65ae2f70f976435d6d360a6a | rpl22_mfa_gb_codon3, rpl23_mfa_gb_codon3, rpl20_mfa_gb_codon3, rpl2_mfa_gb_codon3, rps3_mfa_gb_codon3, rps8_mfa_gb_codon3, rpoA_mfa_gb_codon3, rps11_mfa_gb_codon2, rps19_mfa_gb_codon3, rpoC1_mfa_gb_codon3, rpoB_mfa_gb_codon3, chlN_mfa_gb_codon3, chlL_mfa_gb_codon3, rpl14_mfa_gb_codon3, ndhK_mfa_gb_codon1, rpl16_mfa_gb_codon3, ndhG_mfa_gb_codon2, ndhI_mfa_gb_codon3, ycf4_mfa_gb_codon3, ndhJ_mfa_gb_codon3, ndhA_mfa_gb_codon3, ccsA_mfa_gb_codon3
7 | GTR+I+G | 1603 | b24aad6d0ccf9ffe80496445a8eec090 | rps4_mfa_gb_codon3, petA_mfa_gb_codon3, atpE_mfa_gb_codon3, atpF_mfa_gb_codon3, infA_mfa_gb_codon2, rps2_mfa_gb_codon3, atpA_mfa_gb_codon3
8 | GTR+I+G | 3261 | af1696cc3f3919f4df3fcf6352699f36 | ycf2_mfa_gb_codon3, ycf2_mfa_gb_codon1, ycf2_mfa_gb_codon2
9 | GTR+I+G | 1451 | 9e9d21aa10dadeb5b29fdb6efa404597 | rpl2_mfa_gb_codon2, rps3_mfa_gb_codon2, atpE_mfa_gb_codon2, chlN_mfa_gb_codon2, atpI_mfa_gb_codon1, rps7_mfa_gb_codon2
10 | GTR+I+G | 2001 | 42b2fd4800b8245ea438f83e722cf8a3 | ycf66_mfa_gb_codon1, cemA_mfa_gb_codon3, ndhG_mfa_gb_codon3, rps15_mfa_gb_codon1, rpoC2_mfa_gb_codon1
11 | GTR+I+G | 1873 | 6c770b85965edb052d50bfd5e226c678 | rps7_mfa_gb_codon3, rpl21_mfa_gb_codon3, psbL_mfa_gb_codon3, rps14_mfa_gb_codon1, ccsA_mfa_gb_codon1, rpl36_mfa_gb_codon2, rpoC2_mfa_gb_codon2
12 | GTR+G | 928 | fff44819af8d14fde6b4db132c8ef0ce | ndhH_mfa_gb_codon2, ycf3_mfa_gb_codon1, chlL_mfa_gb_codon1, psaJ_mfa_gb_codon1, psbZ_mfa_gb_codon1, psbJ_mfa_gb_codon1
13 | GTR+I+G | 2917 | e921eac04c02b040714b08a923161282 | ycf3_mfa_gb_codon2, atpA_mfa_gb_codon2, psbL_mfa_gb_codon2, atpB_mfa_gb_codon2, psaB_mfa_gb_codon2, psaA_mfa_gb_codon2, psaC_mfa_gb_codon2, psbE_mfa_gb_codon2, psbE_mfa_gb_codon1, psbF_mfa_gb_codon1
14 | GTR+I+G | 761 | ced1e6fddc2aa6742c2e2b312e7305c0 | ycf3_mfa_gb_codon3, psbD_mfa_gb_codon3, psaC_mfa_gb_codon3, petN_mfa_gb_codon3, psbM_mfa_gb_codon3, psbT_mfa_gb_codon3, ndhE_mfa_gb_codon3, psaJ_mfa_gb_codon3
15 | GTR+I+G | 988 | 83277daffd3fbd7ecc74ce283879d36e | rpl23_mfa_gb_codon2, rps4_mfa_gb_codon2, rps8_mfa_gb_codon2, rpoA_mfa_gb_codon2, rpl32_mfa_gb_codon2, rpoA_mfa_gb_codon1, rpl20_mfa_gb_codon2
16 | GTR+G | 1054 | 154c12e7a76559e500be7be241f5ccaa | rpl16_mfa_gb_codon2, rps12_mfa_gb_codon2, rps12_mfa_gb_codon3, rpl16_mfa_gb_codon1, rps7_mfa_gb_codon1, rps19_mfa_gb_codon1, petA_mfa_gb_codon1, psbM_mfa_gb_codon1, rps11_mfa_gb_codon3

17 | GTR+I+G | 4186 | bb9fa62998536f010786975ab3b3693a | rps19_mfa_gb_codon2, rps11_mfa_gb_codon1, psaA_mfa_gb_codon1, psbC_mfa_gb_codon1, psbN_mfa_gb_codon1, rbcL_mfa_gb_codon1, atpA_mfa_gb_codon1, atpB_mfa_gb_codon1, psbB_mfa_gb_codon1, psaB_mfa_gb_codon1
18 | GTR+I+G | 641 | 9e5a070a1b7ba3051f12feb8373f7a5a | ndhE_mfa_gb_codon2, rps15_mfa_gb_codon2, psbF_mfa_gb_codon3, ccsA_mfa_gb_codon2, ycf12_mfa_gb_codon2, rps14_mfa_gb_codon3
19 | GTR+I+G | 1697 | 47109e327b46035d9f635900401daf8d | psaM_mfa_gb_codon2, ndhG_mfa_gb_codon1, psaM_mfa_gb_codon1, ndhF_mfa_gb_codon1, ycf12_mfa_gb_codon3, ndhC_mfa_gb_codon1, ndhD_mfa_gb_codon3, petG_mfa_gb_codon3, ndhC_mfa_gb_codon3
20 | GTR+G | 1972 | 27f35a427c5bd8e4e21c7541e00e798e | clpP_mfa_gb_codon1, ndhH_mfa_gb_codon3, rpl14_mfa_gb_codon2, chlL_mfa_gb_codon2, psbT_mfa_gb_codon1, petA_mfa_gb_codon2, ndhJ_mfa_gb_codon2, chlB_mfa_gb_codon2
21 | GTR+I+G | 4254 | 328266813408c662f30071b8c0c7f9d8 | psbH_mfa_gb_codon3, psbC_mfa_gb_codon3, ndhH_mfa_gb_codon1, atpB_mfa_gb_codon3, clpP_mfa_gb_codon2, rps18_mfa_gb_codon3, rbcL_mfa_gb_codon3, psbZ_mfa_gb_codon3, atpH_mfa_gb_codon3, psbJ_mfa_gb_codon3, petB_mfa_gb_codon3, psbN_mfa_gb_codon3, chlB_mfa_gb_codon3, psaA_mfa_gb_codon3, petD_mfa_gb_codon3, atpI_mfa_gb_codon3
22 | GTR+G | 534 | 01284e2f240163a49397253bb917a6ab | rpl32_mfa_gb_codon1, rpl23_mfa_gb_codon1, infA_mfa_gb_codon3, rpl33_mfa_gb_codon2, rpl22_mfa_gb_codon1, rpl33_mfa_gb_codon1, rpl20_mfa_gb_codon1
23 | GTR+G | 103 | 639dc8bd84d6e98ecf3cfc7db0192727 | rpl21_mfa_gb_codon2
24 | GTR+I+G | 51 | 4ee832deefd166d2062f5a767e736ad7 | rpl32_mfa_gb_codon3
25 | GTR+I+G | 2353 | e2b21a3c2820d3a6a4578d5bdfd6ce45 | psaI_mfa_gb_codon3, psbK_mfa_gb_codon3, ndhB_mfa_gb_codon1, cemA_mfa_gb_codon1, ndhF_mfa_gb_codon3, ycf66_mfa_gb_codon2, ndhD_mfa_gb_codon2, rpl36_mfa_gb_codon3
26 | GTR+I+G | 905 | d5297f5c017a069c646965057d8ff70f | atpH_mfa_gb_codon1, psbD_mfa_gb_codon1, psbI_mfa_gb_codon1, petB_mfa_gb_codon1, psaC_mfa_gb_codon1, petD_mfa_gb_codon1
27 | GTR+G | 1689 | 79bd0b9fe704082cae80003c7a6336f9 | psbB_mfa_gb_codon2, psbC_mfa_gb_codon2, psbD_mfa_gb_codon2, petB_mfa_gb_codon2, atpH_mfa_gb_codon2, psbF_mfa_gb_codon2, psbJ_mfa_gb_codon2
28 | GTR+I+G | 1900 | eba6ea78114b6a71982d54c5b7764063 | ndhA_mfa_gb_codon2, psbH_mfa_gb_codon2, psbK_mfa_gb_codon2, psbM_mfa_gb_codon2, psbZ_mfa_gb_codon2, psaJ_mfa_gb_codon2, atpI_mfa_gb_codon2, petN_mfa_gb_codon2, ndhB_mfa_gb_codon3, ndhD_mfa_gb_codon1, psaI_mfa_gb_codon2, psbT_mfa_gb_codon2
29 | GTR+G | 909 | f9b058b22c67b2160a1ad4dee7666dba | ndhK_mfa_gb_codon3, ndhF_mfa_gb_codon2, psaI_mfa_gb_codon1
30 | GTR+G | 368 | 7f74bd9349dc18649f09d66c2ee9c3a3 | petG_mfa_gb_codon2, petG_mfa_gb_codon1, petN_mfa_gb_codon1, psbN_mfa_gb_codon2, psbL_mfa_gb_codon1, psbI_mfa_gb_codon2, petD_mfa_gb_codon2
31 | GTR+I+G | 465 | 8be8526f01c01706a7a01762f4dc5fe3 | petL_mfa_gb_codon2, psbA_mfa_gb_codon3, psbE_mfa_gb_codon3
32 | GTR+I+G | 1304 | 6ae4f872ef7413bd71cceba0e70a6456 | petL_mfa_gb_codon3, psaB_mfa_gb_codon3, psbI_mfa_gb_codon3, psbB_mfa_gb_codon3
33 | GTR+I+G | 1182 | 81ed15bd41503814eb201bc035b4ff06 | rbcL_mfa_gb_codon2, psbA_mfa_gb_codon1, psbA_mfa_gb_codon2

Nexus formatted character sets for IQtree

```
#nexus
begin sets;
	charset Subset1 = 32251-33591\3 26435-27753\3 1-3240\3 9906-13386\3 32252-33591\3 16633-16743\3 18949-19257\3 20022-20181\3;
```

charset Subset2 = 2-3240\3 50589-50673\3;
charset Subset3 = 3-3240\3 32253-33591\3 17505-17757\3 20283-20712\3;
charset Subset4 = 27754-29274\3 30124-31455\3 42349-42765\3 31458-32028\3 41866-42348\3 42767-43344\3 16844-17139\3 18160-18375\3 32029-32250\3 5161-5838\3 37858-38148\3 59347-62457\3 3241-5160\3 14368-14913\3 19259-19602\3 14914-15426\3 20182-20280\3 23683-24069\3 24070-24591\3 13387-14022\3 15883-16263\3 17140-17502\3 9100-9903\3;
charset Subset5 = 45493-45576\3 34595-36048\3 16744-16842\3 33592-34593\3 36050-36402\3 18161-18375\3 14915-15426\3 56047-56262\3 56494-56643\3 41867-42348\3 3242-5160\3 59348-62457\3 24071-24591\3 5162-5838\3;
charset Subset6 = 19260-19602\3 19605-19866\3 18657-18948\3 9102-9903\3 13389-14022\3 15885-16263\3 58686-59346\3 16265-16632\3 18378-18654\3 3243-5160\3 59349-62457\3 30126-31455\3 29277-30123\3 17142-17502\3 42766-43344\3 17760-18159\3 40193-40704\3 41868-42348\3 14916-15426\3 42351-42765\3 33594-34593\3 25575-26433\3;
charset Subset7 = 14370-14913\3 43347-44283\3 23685-24069\3 24072-24591\3 32030-32250\3 5163-5838\3 20715-22227\3;
charset Subset8 = 5841-9099\3 5839-9099\3 5840-9099\3;
charset Subset9 = 9101-9903\3 13388-14022\3 23684-24069\3 30125-31455\3 24835-25572\3 15428-15882\3;
charset Subset10 = 20281-20712\3 26436-27753\3 40194-40704\3 17503-17757\3 9904-13386\3;
charset Subset11 = 15429-15882\3 18951-19257\3 56646-56757\3 16843-17139\3 25573-26433\3 20183-20280\3 9905-13386\3;
charset Subset12 = 40706-41865\3 14023-14367\3 29275-30123\3 50467-50586\3 57070-57255\3 56371-56493\3;
charset Subset13 = 14024-14367\3 20714-22227\3 56645-56757\3 22229-23682\3 47915-50115\3 45665-47913\3 50117-50361\3 55676-55926\3 55675-55926\3 55927-56046\3;
charset Subset14 = 14025-14367\3 54669-55674\3 50118-50361\3 45579-45663\3 56760-56850\3 56979-57069\3 37860-38148\3 50469-50586\3;
charset Subset15 = 19604-19866\3 14369-14913\3 15884-16263\3 58685-59346\3 19868-20019\3 58684-59346\3 18656-18948\3;
charset Subset16 = 17759-18159\3 16634-16743\3 16635-16743\3 17758-18159\3 15427-15882\3 18376-18654\3 43345-44283\3 56758-56850\3 16266-16632\3;
charset Subset17 = 18377-18654\3 16264-16632\3 45664-47913\3 53251-54666\3 56851-56976\3 57256-58683\3 20713-22227\3 22228-23682\3 51733-53250\3 47914-50115\3;
charset Subset18 = 37859-38148\3 17504-17757\3 55929-56046\3 25574-26433\3 16745-16842\3 16845-17139\3;
charset Subset19 = 50588-50673\3 40192-40704\3 50587-50673\3 38149-40191\3 16746-16842\3 36049-36402\3 36405-37857\3 45396-45492\3 36051-36402\3;
charset Subset20 = 31456-32028\3 40707-41865\3 17141-17502\3 29276-30123\3 56977-57069\3 43346-44283\3 42350-42765\3 27755-29274\3;
charset Subset21 = 56049-56262\3 53253-54666\3 40705-41865\3 22230-23682\3 31457-32028\3 18162-18375\3 57258-58683\3 57072-57255\3 24594-24834\3 56373-56493\3 44286-44922\3 56853-56976\3 27756-29274\3 45666-47913\3 44925-45393\3 24837-25572\3;
charset Subset22 = 19867-20019\3 19603-19866\3 32031-32250\3 20021-20181\3 19258-19602\3 20020-20181\3 18655-18948\3;
charset Subset23 = 18950-19257\3;
charset Subset24 = 19869-20019\3;
charset Subset25 = 50364-50466\3 56496-56643\3 34594-36048\3 26434-27753\3 38151-40191\3 20282-20712\3 36404-37857\3 20184-20280\3;
charset Subset26 = 24592-24834\3 54667-55674\3 56263-56370\3 44284-44922\3 50116-50361\3 44923-45393\3;

charset Subset27 = 51734-53250\3 53252-54666\3 54668-55674\3 44285-44922\3 24593-24834\3 55928-56046\3 56372-56493\3;
charset Subset28 = 33593-34593\3 56048-56262\3 56495-56643\3 56759-56850\3 57071-57255\3 50468-50586\3 24836-25572\3 45578-45663\3 34596-36048\3 36403-37857\3 50363-50466\3 56978-57069\3;
charset Subset29 = 42768-43344\3 38150-40191\3 50362-50466\3;
charset Subset30 = 45395-45492\3 45394-45492\3 45577-45663\3 56852-56976\3 56644-56757\3 56264-56370\3 44924-45393\3;
charset Subset31 = 45494-45576\3 50676-51732\3 55677-55926\3;
charset Subset32 = 45495-45576\3 47916-50115\3 56265-56370\3 51735-53250\3;
charset Subset33 = 57257-58683\3 50674-51732\3 50675-51732\3;
charpartition PartitionFinder = GTR+I+G:Subset1, GTR+I+G:Subset2, GTR+I+G:Subset3, GTR+I+G:Subset4, GTR+I+G:Subset5, GTR+I+G:Subset6, GTR+I+G:Subset7, GTR+I+G:Subset8, GTR+I+G:Subset9, GTR+I+G:Subset10, GTR+I+G:Subset11, GTR+G:Subset12, GTR+I+G:Subset13, GTR+I+G:Subset14, GTR+I+G:Subset15, GTR+G:Subset16, GTR+I+G:Subset17, GTR+I+G:Subset18, GTR+I+G:Subset19, GTR+G:Subset20, GTR+I+G:Subset21, GTR+G:Subset22, GTR+G:Subset23, GTR+I+G:Subset24, GTR+I+G:Subset25, GTR+I+G:Subset26, GTR+G:Subset27, GTR+I+G:Subset28, GTR+G:Subset29, GTR+G:Subset30, GTR+I+G:Subset31, GTR+I+G:Subset32, GTR+I+G:Subset33;
end;
MrBayes block for partition definitions
begin mrbayes;
charset Subset1 = 32251-33591\3 26435-27753\3 1-3240\3 9906-13386\3 32252-33591\3 16633-16743\3 18949-19257\3 20022-20181\3;
charset Subset2 = 2-3240\3 50589-50673\3;
charset Subset3 = 3-3240\3 32253-33591\3 17505-17757\3 20283-20712\3;
charset Subset4 = 27754-29274\3 30124-31455\3 42349-42765\3 31458-32028\3 41866-42348\3 42767-43344\3 16844-17139\3 18160-18375\3 32029-32250\3 5161-5838\3 37858-38148\3 59347-62457\3 3241-5160\3 14368-14913\3 19259-19602\3 14914-15426\3 20182-20280\3 23683-24069\3 24070-24591\3 13387-14022\3 15883-16263\3 17140-17502\3 9100-9903\3;
charset Subset5 = 45493-45576\3 34595-36048\3 16744-16842\3 33592-34593\3 36050-36402\3 18161-18375\3 14915-15426\3 56047-56262\3 56494-56643\3 41867-42348\3 3242-5160\3 59348-62457\3 24071-24591\3 5162-5838\3;
charset Subset6 = 19260-19602\3 19605-19866\3 18657-18948\3 9102-9903\3 13389-14022\3 15885-16263\3 58686-59346\3 16265-16632\3 18378-18654\3 3243-5160\3 59349-62457\3 30126-31455\3 29277-30123\3 17142-17502\3 42766-43344\3 17760-18159\3 40193-40704\3 41868-42348\3 14916-15426\3 42351-42765\3 33594-34593\3 25575-26433\3;
charset Subset7 = 14370-14913\3 43347-44283\3 23685-24069\3 24072-24591\3 32030-32250\3 5163-5838\3 20715-22227\3;
charset Subset8 = 5841-9099\3 5839-9099\3 5840-9099\3;
charset Subset9 = 9101-9903\3 13388-14022\3 23684-24069\3 30125-31455\3 24835-25572\3 15428-15882\3;
charset Subset10 = 20281-20712\3 26436-27753\3 40194-40704\3 17503-17757\3 9904-13386\3;
charset Subset11 = 15429-15882\3 18951-19257\3 56646-56757\3 16843-17139\3 25573-26433\3 20183-20280\3 9905-13386\3;
charset Subset12 = 40706-41865\3 14023-14367\3 29275-30123\3 50467-50586\3 57070-57255\3 56371-56493\3;
charset Subset13 = 14024-14367\3 20714-22227\3 56645-56757\3 22229-23682\3 47915-50115\3 45665-47913\3 50117-50361\3 55676-55926\3 55675-55926\3 55927-56046\3;
charset Subset14 = 14025-14367\3 54669-55674\3 50118-50361\3 45579-45663\3 56760-56850\3 56979-57069\3 37860-38148\3 50469-50586\3;
charset Subset15 = 19604-19866\3 14369-14913\3 15884-16263\3 58685-59346\3 19868-20019\3 58684-59346\3 18656-18948\3;

charset Subset16 = 17759-18159\3 16634-16743\3 16635-16743\3 17758-18159\3 15427-15882\3 18376-18654\3 43345-44283\3 56758-56850\3 16266-16632\3;

charset Subset17 = 18377-18654\3 16264-16632\3 45664-47913\3 53251-54666\3 56851-56976\3 57256-58683\3 20713-22227\3 22228-23682\3 51733-53250\3 47914-50115\3;

charset Subset18 = 37859-38148\3 17504-17757\3 55929-56046\3 25574-26433\3 16745-16842\3 16845-17139\3;

charset Subset19 = 50588-50673\3 40192-40704\3 50587-50673\3 38149-40191\3 16746-16842\3 36049-36402\3 36405-37857\3 45396-45492\3 36051-36402\3;

charset Subset20 = 31456-32028\3 40707-41865\3 17141-17502\3 29276-30123\3 56977-57069\3 43346-44283\3 42350-42765\3 27755-29274\3;

charset Subset21 = 56049-56262\3 53253-54666\3 40705-41865\3 22230-23682\3 31457-32028\3 18162-18375\3 57258-58683\3 57072-57255\3 24594-24834\3 56373-56493\3 44286-44922\3 56853-56976\3 27756-29274\3 45666-47913\3 44925-45393\3 24837-25572\3;

charset Subset22 = 19867-20019\3 19603-19866\3 32031-32250\3 20021-20181\3 19258-19602\3 20020-20181\3 18655-18948\3;

charset Subset23 = 18950-19257\3;

charset Subset24 = 19869-20019\3;

charset Subset25 = 50364-50466\3 56496-56643\3 34594-36048\3 26434-27753\3 38151-40191\3 20282-20712\3 36404-37857\3 20184-20280\3;

charset Subset26 = 24592-24834\3 54667-55674\3 56263-56370\3 44284-44922\3 50116-50361\3 44923-45393\3;

charset Subset27 = 51734-53250\3 53252-54666\3 54668-55674\3 44285-44922\3 24593-24834\3 55928-56046\3 56372-56493\3;

charset Subset28 = 33593-34593\3 56048-56262\3 56495-56643\3 56759-56850\3 57071-57255\3 50468-50586\3 24836-25572\3 45578-45663\3 34596-36048\3 36403-37857\3 50363-50466\3 56978-57069\3;

charset Subset29 = 42768-43344\3 38150-40191\3 50362-50466\3;

charset Subset30 = 45395-45492\3 45394-45492\3 45577-45663\3 56852-56976\3 56644-56757\3 56264-56370\3 44924-45393\3;

charset Subset31 = 45494-45576\3 50676-51732\3 55677-55926\3;

charset Subset32 = 45495-45576\3 47916-50115\3 56265-56370\3 51735-53250\3;

charset Subset33 = 57257-58683\3 50674-51732\3 50675-51732\3;

partition PartitionFinder = 33:Subset1, Subset2, Subset3, Subset4, Subset5, Subset6, Subset7, Subset8, Subset9, Subset10, Subset11, Subset12, Subset13, Subset14, Subset15, Subset16, Subset17, Subset18, Subset19, Subset20, Subset21, Subset22, Subset23, Subset24, Subset25, Subset26, Subset27, Subset28, Subset29, Subset30, Subset31, Subset32, Subset33;

set partition=PartitionFinder;

lset applyto=(1) nst=6 rates=invgamma;
lset applyto=(2) nst=6 rates=invgamma;
lset applyto=(3) nst=6 rates=invgamma;
lset applyto=(4) nst=6 rates=invgamma;
lset applyto=(5) nst=6 rates=invgamma;
lset applyto=(6) nst=6 rates=invgamma;
lset applyto=(7) nst=6 rates=invgamma;
lset applyto=(8) nst=6 rates=invgamma;
lset applyto=(9) nst=6 rates=invgamma;
lset applyto=(10) nst=6 rates=invgamma;
lset applyto=(11) nst=6 rates=invgamma;
lset applyto=(12) nst=6 rates=gamma;
lset applyto=(13) nst=6 rates=invgamma;

```
	lset applyto=(14) nst=6 rates=invgamma;
	lset applyto=(15) nst=6 rates=invgamma;
	lset applyto=(16) nst=6 rates=gamma;
	lset applyto=(17) nst=6 rates=invgamma;
	lset applyto=(18) nst=6 rates=invgamma;
	lset applyto=(19) nst=6 rates=invgamma;
	lset applyto=(20) nst=6 rates=gamma;
	lset applyto=(21) nst=6 rates=invgamma;
	lset applyto=(22) nst=6 rates=gamma;
	lset applyto=(23) nst=6 rates=gamma;
	lset applyto=(24) nst=6 rates=invgamma;
	lset applyto=(25) nst=6 rates=invgamma;
	lset applyto=(26) nst=6 rates=invgamma;
	lset applyto=(27) nst=6 rates=gamma;
	lset applyto=(28) nst=6 rates=invgamma;
	lset applyto=(29) nst=6 rates=gamma;
	lset applyto=(30) nst=6 rates=gamma;
	lset applyto=(31) nst=6 rates=invgamma;
	lset applyto=(32) nst=6 rates=invgamma;
	lset applyto=(33) nst=6 rates=invgamma;
	prset applyto=(all) ratepr=variable;
	unlink statefreq=(all) revmat=(all) shape=(all) pinvar=(all) tratio=(all);
end;
```

**Supplementary Files S2.** Best partition schemes of PartitionFinder2 used in divergence time estimation and ancestral state reconstruction.

```
# DATA BLOCKS #
[data_blocks]
rpoC1_mfa_gb_codon1=1-1911\3;
rpoC1_mfa_gb_codon2=2-1911\3;
rpoC1_mfa_gb_codon3=3-1911\3;
ycf1_mfa_gb_codon1=1912-5133\3;
ycf1_mfa_gb_codon2=1913-5133\3;
ycf1_mfa_gb_codon3=1914-5133\3;
rpl2_mfa_gb_codon1=5134-5943\3;
rpl2_mfa_gb_codon2=5135-5943\3;
rpl2_mfa_gb_codon3=5136-5943\3;
rpoC2_mfa_gb_codon1=5944-9315\3;
rpoC2_mfa_gb_codon2=5945-9315\3;
rpoC2_mfa_gb_codon3=5946-9315\3;
rps2_mfa_gb_codon1=9316-9990\3;
rps2_mfa_gb_codon2=9317-9990\3;
rps2_mfa_gb_codon3=9318-9990\3;
ycf2_mfa_gb_codon1=9991-13113\3;
ycf2_mfa_gb_codon2=9992-13113\3;
ycf2_mfa_gb_codon3=9993-13113\3;
rps3_mfa_gb_codon1=13114-13761\3;
rps3_mfa_gb_codon2=13115-13761\3;
rps3_mfa_gb_codon3=13116-13761\3;
ycf3_mfa_gb_codon1=13762-14106\3;
ycf3_mfa_gb_codon2=13763-14106\3;
ycf3_mfa_gb_codon3=13764-14106\3;
rps4_mfa_gb_codon1=14107-14661\3;
```

rps4_mfa_gb_codon2=14108-14661\3;
rps4_mfa_gb_codon3=14109-14661\3;
ycf4_mfa_gb_codon1=14662-15192\3;
ycf4_mfa_gb_codon2=14663-15192\3;
ycf4_mfa_gb_codon3=14664-15192\3;
rps7_mfa_gb_codon1=15193-15633\3;
rps7_mfa_gb_codon2=15194-15633\3;
rps7_mfa_gb_codon3=15195-15633\3;
rps8_mfa_gb_codon1=15634-16026\3;
rps8_mfa_gb_codon2=15635-16026\3;
rps8_mfa_gb_codon3=15636-16026\3;
rps11_mfa_gb_codon1=16027-16392\3;
rps11_mfa_gb_codon2=16028-16392\3;
rps11_mfa_gb_codon3=16029-16392\3;
rps12_mfa_gb_codon1=16393-16503\3;
rps12_mfa_gb_codon2=16394-16503\3;
rps12_mfa_gb_codon3=16395-16503\3;
ycf12_mfa_gb_codon1=16504-16602\3;
ycf12_mfa_gb_codon2=16505-16602\3;
ycf12_mfa_gb_codon3=16506-16602\3;
rpl14_mfa_gb_codon1=16603-16968\3;
rpl14_mfa_gb_codon2=16604-16968\3;
rpl14_mfa_gb_codon3=16605-16968\3;
rps14_mfa_gb_codon1=16969-17265\3;
rps14_mfa_gb_codon2=16970-17265\3;
rps14_mfa_gb_codon3=16971-17265\3;
rps15_mfa_gb_codon1=17266-17517\3;
rps15_mfa_gb_codon2=17267-17517\3;
rps15_mfa_gb_codon3=17268-17517\3;
rpl16_mfa_gb_codon1=17518-17913\3;
rpl16_mfa_gb_codon2=17519-17913\3;
rpl16_mfa_gb_codon3=17520-17913\3;
rps18_mfa_gb_codon1=17914-18129\3;
rps18_mfa_gb_codon2=17915-18129\3;
rps18_mfa_gb_codon3=17916-18129\3;
rps19_mfa_gb_codon1=18130-18408\3;
rps19_mfa_gb_codon2=18131-18408\3;
rps19_mfa_gb_codon3=18132-18408\3;
rpl20_mfa_gb_codon1=18409-18720\3;
rpl20_mfa_gb_codon2=18410-18720\3;
rpl20_mfa_gb_codon3=18411-18720\3;
rpl21_mfa_gb_codon1=18721-19032\3;
rpl21_mfa_gb_codon2=18722-19032\3;
rpl21_mfa_gb_codon3=18723-19032\3;
rpl22_mfa_gb_codon1=19033-19374\3;
rpl22_mfa_gb_codon2=19034-19374\3;
rpl22_mfa_gb_codon3=19035-19374\3;
rpl23_mfa_gb_codon1=19375-19638\3;
rpl23_mfa_gb_codon2=19376-19638\3;
rpl23_mfa_gb_codon3=19377-19638\3;
rpl32_mfa_gb_codon1=19639-19791\3;

rpl32_mfa_gb_codon2=19640-19791\3;
rpl32_mfa_gb_codon3=19641-19791\3;
rpl33_mfa_gb_codon1=19792-19962\3;
rpl33_mfa_gb_codon2=19793-19962\3;
rpl33_mfa_gb_codon3=19794-19962\3;
rpl36_mfa_gb_codon1=19963-20073\3;
rpl36_mfa_gb_codon2=19964-20073\3;
rpl36_mfa_gb_codon3=19965-20073\3;
ycf66_mfa_gb_codon1=20074-20487\3;
ycf66_mfa_gb_codon2=20075-20487\3;
ycf66_mfa_gb_codon3=20076-20487\3;
atpA_mfa_gb_codon1=20488-22002\3;
atpA_mfa_gb_codon2=20489-22002\3;
atpA_mfa_gb_codon3=20490-22002\3;
atpB_mfa_gb_codon1=22003-23457\3;
atpB_mfa_gb_codon2=22004-23457\3;
atpB_mfa_gb_codon3=22005-23457\3;
atpE_mfa_gb_codon1=23458-23862\3;
atpE_mfa_gb_codon2=23459-23862\3;
atpE_mfa_gb_codon3=23460-23862\3;
atpF_mfa_gb_codon1=23863-24384\3;
atpF_mfa_gb_codon2=23864-24384\3;
atpF_mfa_gb_codon3=23865-24384\3;
atpH_mfa_gb_codon1=24385-24627\3;
atpH_mfa_gb_codon2=24386-24627\3;
atpH_mfa_gb_codon3=24387-24627\3;
atpI_mfa_gb_codon1=24628-25365\3;
atpI_mfa_gb_codon2=24629-25365\3;
atpI_mfa_gb_codon3=24630-25365\3;
ccsA_mfa_gb_codon1=25366-26223\3;
ccsA_mfa_gb_codon2=25367-26223\3;
ccsA_mfa_gb_codon3=25368-26223\3;
cemA_mfa_gb_codon1=26224-27423\3;
cemA_mfa_gb_codon2=26225-27423\3;
cemA_mfa_gb_codon3=26226-27423\3;
chlB_mfa_gb_codon1=27424-28944\3;
chlB_mfa_gb_codon2=27425-28944\3;
chlB_mfa_gb_codon3=27426-28944\3;
chlL_mfa_gb_codon1=28945-29796\3;
chlL_mfa_gb_codon2=28946-29796\3;
chlL_mfa_gb_codon3=28947-29796\3;
chlN_mfa_gb_codon1=29797-31113\3;
chlN_mfa_gb_codon2=29798-31113\3;
chlN_mfa_gb_codon3=29799-31113\3;
clpP_mfa_gb_codon1=31114-31698\3;
clpP_mfa_gb_codon2=31115-31698\3;
clpP_mfa_gb_codon3=31116-31698\3;
infA_mfa_gb_codon1=31699-31929\3;
infA_mfa_gb_codon2=31700-31929\3;
infA_mfa_gb_codon3=31701-31929\3;
matK_mfa_gb_codon1=31930-33261\3;
matK_mfa_gb_codon2=31931-33261\3;

matK_mfa_gb_codon3=31932-33261\3;
ndhA_mfa_gb_codon1=33262-34224\3;
ndhA_mfa_gb_codon2=33263-34224\3;
ndhA_mfa_gb_codon3=33264-34224\3;
ndhB_mfa_gb_codon1=34225-35679\3;
ndhB_mfa_gb_codon2=34226-35679\3;
ndhB_mfa_gb_codon3=34227-35679\3;
ndhC_mfa_gb_codon1=35680-36036\3;
ndhC_mfa_gb_codon2=35681-36036\3;
ndhC_mfa_gb_codon3=35682-36036\3;
ndhD_mfa_gb_codon1=36037-37494\3;
ndhD_mfa_gb_codon2=36038-37494\3;
ndhD_mfa_gb_codon3=36039-37494\3;
ndhE_mfa_gb_codon1=37495-37791\3;
ndhE_mfa_gb_codon2=37496-37791\3;
ndhE_mfa_gb_codon3=37497-37791\3;
ndhF_mfa_gb_codon1=37792-39837\3;
ndhF_mfa_gb_codon2=37793-39837\3;
ndhF_mfa_gb_codon3=37794-39837\3;
ndhG_mfa_gb_codon1=39838-40368\3;
ndhG_mfa_gb_codon2=39839-40368\3;
ndhG_mfa_gb_codon3=39840-40368\3;
ndhH_mfa_gb_codon1=40369-41541\3;
ndhH_mfa_gb_codon2=40370-41541\3;
ndhH_mfa_gb_codon3=40371-41541\3;
ndhI_mfa_gb_codon1=41542-42027\3;
ndhI_mfa_gb_codon2=41543-42027\3;
ndhI_mfa_gb_codon3=41544-42027\3;
ndhJ_mfa_gb_codon1=42028-42498\3;
ndhJ_mfa_gb_codon2=42029-42498\3;
ndhJ_mfa_gb_codon3=42030-42498\3;
ndhK_mfa_gb_codon1=42499-43068\3;
ndhK_mfa_gb_codon2=42500-43068\3;
ndhK_mfa_gb_codon3=42501-43068\3;
petA_mfa_gb_codon1=43069-44010\3;
petA_mfa_gb_codon2=43070-44010\3;
petA_mfa_gb_codon3=43071-44010\3;
petB_mfa_gb_codon1=44011-44646\3;
petB_mfa_gb_codon2=44012-44646\3;
petB_mfa_gb_codon3=44013-44646\3;
petD_mfa_gb_codon1=44647-45117\3;
petD_mfa_gb_codon2=44648-45117\3;
petD_mfa_gb_codon3=44649-45117\3;
petG_mfa_gb_codon1=45118-45216\3;
petG_mfa_gb_codon2=45119-45216\3;
petG_mfa_gb_codon3=45120-45216\3;
petL_mfa_gb_codon1=45217-45306\3;
petL_mfa_gb_codon2=45218-45306\3;
petL_mfa_gb_codon3=45219-45306\3;
petN_mfa_gb_codon1=45307-45396\3;
petN_mfa_gb_codon2=45308-45396\3;

petN_mfa_gb_codon3=45309-45396\3;
psaA_mfa_gb_codon1=45397-47646\3;
psaA_mfa_gb_codon2=45398-47646\3;
psaA_mfa_gb_codon3=45399-47646\3;
psaB_mfa_gb_codon1=47647-49848\3;
psaB_mfa_gb_codon2=47648-49848\3;
psaB_mfa_gb_codon3=47649-49848\3;
psaC_mfa_gb_codon1=49849-50094\3;
psaC_mfa_gb_codon2=49850-50094\3;
psaC_mfa_gb_codon3=49851-50094\3;
psaI_mfa_gb_codon1=50095-50199\3;
psaI_mfa_gb_codon2=50096-50199\3;
psaI_mfa_gb_codon3=50097-50199\3;
psaJ_mfa_gb_codon1=50200-50319\3;
psaJ_mfa_gb_codon2=50201-50319\3;
psaJ_mfa_gb_codon3=50202-50319\3;
psaM_mfa_gb_codon1=50320-50409\3;
psaM_mfa_gb_codon2=50321-50409\3;
psaM_mfa_gb_codon3=50322-50409\3;
psbA_mfa_gb_codon1=50410-51468\3;
psbA_mfa_gb_codon2=50411-51468\3;
psbA_mfa_gb_codon3=50412-51468\3;
psbB_mfa_gb_codon1=51469-52986\3;
psbB_mfa_gb_codon2=51470-52986\3;
psbB_mfa_gb_codon3=51471-52986\3;
psbC_mfa_gb_codon1=52987-54402\3;
psbC_mfa_gb_codon2=52988-54402\3;
psbC_mfa_gb_codon3=52989-54402\3;
psbD_mfa_gb_codon1=54403-55410\3;
psbD_mfa_gb_codon2=54404-55410\3;
psbD_mfa_gb_codon3=54405-55410\3;
psbE_mfa_gb_codon1=55411-55662\3;
psbE_mfa_gb_codon2=55412-55662\3;
psbE_mfa_gb_codon3=55413-55662\3;
psbF_mfa_gb_codon1=55663-55782\3;
psbF_mfa_gb_codon2=55664-55782\3;
psbF_mfa_gb_codon3=55665-55782\3;
psbH_mfa_gb_codon1=55783-55998\3;
psbH_mfa_gb_codon2=55784-55998\3;
psbH_mfa_gb_codon3=55785-55998\3;
psbI_mfa_gb_codon1=55999-56109\3;
psbI_mfa_gb_codon2=56000-56109\3;
psbI_mfa_gb_codon3=56001-56109\3;
psbJ_mfa_gb_codon1=56110-56232\3;
psbJ_mfa_gb_codon2=56111-56232\3;
psbJ_mfa_gb_codon3=56112-56232\3;
psbK_mfa_gb_codon1=56233-56382\3;
psbK_mfa_gb_codon2=56234-56382\3;
psbK_mfa_gb_codon3=56235-56382\3;
psbL_mfa_gb_codon1=56383-56496\3;
psbL_mfa_gb_codon2=56384-56496\3;
psbL_mfa_gb_codon3=56385-56496\3;

psbM_mfa_gb_codon1=56497-56586\3;
psbM_mfa_gb_codon2=56498-56586\3;
psbM_mfa_gb_codon3=56499-56586\3;
psbN_mfa_gb_codon1=56587-56712\3;
psbN_mfa_gb_codon2=56588-56712\3;
psbN_mfa_gb_codon3=56589-56712\3;
psbT_mfa_gb_codon1=56713-56808\3;
psbT_mfa_gb_codon2=56714-56808\3;
psbT_mfa_gb_codon3=56715-56808\3;
psbZ_mfa_gb_codon1=56809-56994\3;
psbZ_mfa_gb_codon2=56810-56994\3;
psbZ_mfa_gb_codon3=56811-56994\3;
rbcL_mfa_gb_codon1=56995-58422\3;
rbcL_mfa_gb_codon2=56996-58422\3;
rbcL_mfa_gb_codon3=56997-58422\3;
rpoA_mfa_gb_codon1=58423-59085\3;
rpoA_mfa_gb_codon2=58424-59085\3;
rpoA_mfa_gb_codon3=58425-59085\3;
rpoB_mfa_gb_codon1=59086-62193\3;
rpoB_mfa_gb_codon2=59087-62193\3;
rpoB_mfa_gb_codon3=59088-62193\3;
Best partitioning scheme
Subset | Best Model | # sites | subset id | Partition names
1 | GTR+I+G | 4277 | 3e2394ea5690079bac037cecb576d4e4 | rpl22_mfa_gb_codon2, ycf4_mfa_gb_codon1, psaI_mfa_gb_codon1, rpl32_mfa_gb_codon2, ycf12_mfa_gb_codon1, rpoA_mfa_gb_codon1, rps8_mfa_gb_codon2, infA_mfa_gb_codon1, atpF_mfa_gb_codon1, ndhE_mfa_gb_codon1, rps2_mfa_gb_codon1, atpE_mfa_gb_codon1, rps3_mfa_gb_codon1, rpl20_mfa_gb_codon1, rps8_mfa_gb_codon1, rpl2_mfa_gb_codon1, rpl14_mfa_gb_codon1, rps4_mfa_gb_codon1, rpoC1_mfa_gb_codon1, rpoB_mfa_gb_codon1, rpl20_mfa_gb_codon2
2 | GTR+I+G | 3370 | 0b3c50e37e8d99ae9d193b186a77a1ce | rpl23_mfa_gb_codon2, rpoA_mfa_gb_codon2, rps4_mfa_gb_codon2, atpE_mfa_gb_codon2, psbT_mfa_gb_codon2, psaI_mfa_gb_codon2, rps2_mfa_gb_codon2, atpF_mfa_gb_codon2, rpoB_mfa_gb_codon2, ndhI_mfa_gb_codon2, ndhC_mfa_gb_codon2, rps18_mfa_gb_codon2, rpoC1_mfa_gb_codon2, psbH_mfa_gb_codon1, ycf4_mfa_gb_codon2
3 | GTR+I+G | 7542 | c08660c640f670b2e5a5469a5cabc44f | ycf4_mfa_gb_codon3, psbI_mfa_gb_codon3, chlL_mfa_gb_codon3, ndhI_mfa_gb_codon3, atpA_mfa_gb_codon3, ndhA_mfa_gb_codon3, ndhG_mfa_gb_codon3, rpl16_mfa_gb_codon3, rps11_mfa_gb_codon2, rpl14_mfa_gb_codon3, ndhK_mfa_gb_codon3, rpl23_mfa_gb_codon3, rpl20_mfa_gb_codon3, rps19_mfa_gb_codon3, rpoB_mfa_gb_codon3, rpoC1_mfa_gb_codon3, rpl21_mfa_gb_codon2, ndhD_mfa_gb_codon3, rps15_mfa_gb_codon3, ycf66_mfa_gb_codon3, psaM_mfa_gb_codon1, rpl36_mfa_gb_codon3, rpl22_mfa_gb_codon3, rps8_mfa_gb_codon3, rps3_mfa_gb_codon3, rpl2_mfa_gb_codon3, cemA_mfa_gb_codon1, rpoA_mfa_gb_codon3, rpoC2_mfa_gb_codon3
4 | GTR+I+G | 3189 | 95cbb7b48c53c8eadf8f9495c4262fce | ycf2_mfa_gb_codon3, ycf1_mfa_gb_codon1, ycf1_mfa_gb_codon3
5 | GTR+I+G | 3600 | 2c55e6aca544e91c4497ec5ecebf5410 | ycf1_mfa_gb_codon2, ycf2_mfa_gb_codon1, ycf2_mfa_gb_codon2, matK_mfa_gb_codon1
6 | GTR+I+G | 2396 | aa4bbeed754f05de52eeab894c0bf076 | ndhK_mfa_gb_codon2, rpl2_mfa_gb_codon2, psaJ_mfa_gb_codon1, atpI_mfa_gb_codon1, chlN_mfa_gb_codon1, ndhJ_mfa_gb_codon1, chlB_mfa_gb_codon1, clpP_mfa_gb_codon3, ndhI_mfa_gb_codon1, ndhK_mfa_gb_codon1
7 | GTR+I+G | 1124 | 9d8dcbf6580b65fdb11f76c7f1d4325c | rpoC2_mfa_gb_codon1

8 | GTR+I+G | 4356 | 4a99c8f9a3e4258b30984c7058b80c4f | ndhG_mfa_gb_codon2, petL_mfa_gb_codon2, ndhC_mfa_gb_codon1, ndhF_mfa_gb_codon1, psaM_mfa_gb_codon3, ndhG_mfa_gb_codon1, cemA_mfa_gb_codon2, rps14_mfa_gb_codon1, rpl21_mfa_gb_codon3, rps7_mfa_gb_codon3, rpoC2_mfa_gb_codon2, rpl32_mfa_gb_codon1, psaM_mfa_gb_codon2, ycf12_mfa_gb_codon2, rps14_mfa_gb_codon3, ccsA_mfa_gb_codon1, chlN_mfa_gb_codon3, rpl33_mfa_gb_codon1, rps15_mfa_gb_codon2, psbL_mfa_gb_codon3, psbF_mfa_gb_codon3, ycf12_mfa_gb_codon3, infA_mfa_gb_codon2
9 | GTR+I+G | 2065 | 5a373c5a0c0f1190db7dae17a0fb0953 | atpB_mfa_gb_codon3, rpl33_mfa_gb_codon2, rps2_mfa_gb_codon3, ndhH_mfa_gb_codon3, rps4_mfa_gb_codon3, atpE_mfa_gb_codon3, atpF_mfa_gb_codon3, ndhE_mfa_gb_codon3, petA_mfa_gb_codon3
10 | GTR+I+G | 890 | b342eb2df66a052d36af64d53bad56ba | rps3_mfa_gb_codon2, rps14_mfa_gb_codon2, rpl33_mfa_gb_codon3, rps18_mfa_gb_codon1, rpl36_mfa_gb_codon1, rps7_mfa_gb_codon1, rps19_mfa_gb_codon1, rpl16_mfa_gb_codon1, rps12_mfa_gb_codon3
11 | GTR+G | 1890 | 447d118e8e3ebd3080fbca53e53a6186 | rps12_mfa_gb_codon2, rpl16_mfa_gb_codon2, psbJ_mfa_gb_codon1, psbZ_mfa_gb_codon1, chlL_mfa_gb_codon1, ycf3_mfa_gb_codon1, rps11_mfa_gb_codon3, psbM_mfa_gb_codon1, petA_mfa_gb_codon1, ndhH_mfa_gb_codon1, rps11_mfa_gb_codon1, rps19_mfa_gb_codon2, rps7_mfa_gb_codon2
12 | GTR+I+G | 2909 | 21db13f4542031a4d95982b46ced59cf | ycf3_mfa_gb_codon2, atpA_mfa_gb_codon2, psbL_mfa_gb_codon2, psbT_mfa_gb_codon1, atpB_mfa_gb_codon2, psbE_mfa_gb_codon1, psbE_mfa_gb_codon2, psaC_mfa_gb_codon2, psaA_mfa_gb_codon2, psaB_mfa_gb_codon2
13 | GTR+I+G | 563 | 4ea8ef1844a25ce95a5374066390bb34 | ycf3_mfa_gb_codon3, psbD_mfa_gb_codon3, psaC_mfa_gb_codon3, petN_mfa_gb_codon3
14 | GTR+I+G | 2223 | 4c70d0bb37d788e78c84bfb740b0577f | psbM_mfa_gb_codon3, cemA_mfa_gb_codon3, ccsA_mfa_gb_codon2, ndhC_mfa_gb_codon3, petG_mfa_gb_codon3, rps12_mfa_gb_codon1, infA_mfa_gb_codon3, rpl21_mfa_gb_codon1, matK_mfa_gb_codon2, rps15_mfa_gb_codon1, psbT_mfa_gb_codon3, chlN_mfa_gb_codon2, ycf66_mfa_gb_codon1
15 | GTR+G | 1849 | 9b632bf5ab8ebdd28ee9d13882720c8e | petA_mfa_gb_codon2, ndhJ_mfa_gb_codon2, rpl14_mfa_gb_codon2, chlB_mfa_gb_codon2, psbI_mfa_gb_codon2, rpl36_mfa_gb_codon2, ndhH_mfa_gb_codon2, chlL_mfa_gb_codon2
16 | GTR+I+G | 2055 | 14fa3d6e9eeded0c8f200b56c4b7f054 | ycf66_mfa_gb_codon2, ndhF_mfa_gb_codon3, clpP_mfa_gb_codon2, ccsA_mfa_gb_codon3, psbH_mfa_gb_codon3, rps18_mfa_gb_codon3, psaI_mfa_gb_codon3, psaJ_mfa_gb_codon3, ndhB_mfa_gb_codon1, psbK_mfa_gb_codon3
17 | GTR+G | 202 | 799d52f8c8599bbcc62825a436824104 | rpl23_mfa_gb_codon1, rpl22_mfa_gb_codon1
18 | GTR+I+G | 51 | 3d556566558b8ddb9946782779bd715a | rpl32_mfa_gb_codon3
19 | GTR+I+G | 3970 | 35956af3871c3ebabf777393a43640d4 | psaA_mfa_gb_codon1, psbC_mfa_gb_codon1, psbN_mfa_gb_codon1, rbcL_mfa_gb_codon1, atpA_mfa_gb_codon1, atpB_mfa_gb_codon1, psaB_mfa_gb_codon1, psbB_mfa_gb_codon1
20 | GTR+I+G | 945 | 11f3dad9067eeeb8d4505a7067f18e23 | psbD_mfa_gb_codon1, psbI_mfa_gb_codon1, psbF_mfa_gb_codon1, petB_mfa_gb_codon1, atpH_mfa_gb_codon1, petD_mfa_gb_codon1, psaC_mfa_gb_codon1
21 | GTR+G | 1925 | 68accf799957954b99e614960345f9d1 | petD_mfa_gb_codon2, psbL_mfa_gb_codon1, psbN_mfa_gb_codon2, petB_mfa_gb_codon2, psbJ_mfa_gb_codon2, atpH_mfa_gb_codon2, psbF_mfa_gb_codon2, psbD_mfa_gb_codon2, psbC_mfa_gb_codon2, psbB_mfa_gb_codon2
22 | GTR+I+G | 3046 | 8bd54683fd6ab528a4adf5c631db933c | psbC_mfa_gb_codon3, psaA_mfa_gb_codon3, chlB_mfa_gb_codon3, atpI_mfa_gb_codon3, petD_mfa_gb_codon3, rbcL_mfa_gb_codon3, psbZ_mfa_gb_codon3, atpH_mfa_gb_codon3, psbJ_mfa_gb_codon3, petB_mfa_gb_codon3, psbN_mfa_gb_codon3

23 | GTR+G | 2212 | b1c7fac0493ca340fffc81363367e5d9 | petG_mfa_gb_codon2, petG_mfa_gb_codon1, petN_mfa_gb_codon1, petN_mfa_gb_codon2, psbZ_mfa_gb_codon2, psbH_mfa_gb_codon2, psbK_mfa_gb_codon2, psaJ_mfa_gb_codon2, psbM_mfa_gb_codon2, ndhD_mfa_gb_codon2, ndhE_mfa_gb_codon2, ndhB_mfa_gb_codon3, clpP_mfa_gb_codon1, atpI_mfa_gb_codon2, ndhA_mfa_gb_codon2
24 | GTR+I+G | 444 | e07cf2c4777df35f84da71839c291df0 | matK_mfa_gb_codon3
25 | GTR+I+G | 2054 | 00347a339583bd3d15f98f9a01e1e628 | ndhF_mfa_gb_codon2, ndhA_mfa_gb_codon1, psbK_mfa_gb_codon1, petL_mfa_gb_codon1, ndhB_mfa_gb_codon2, ndhD_mfa_gb_codon1
26 | GTR+I+G | 1427 | 340099cdd59bc9818e4a43b974af5289 | petL_mfa_gb_codon3, psbB_mfa_gb_codon3, psaB_mfa_gb_codon3, ndhJ_mfa_gb_codon3
27 | GTR+I+G | 1182 | dc3120cd362f07e0bf3cc80a1076ebe2 | rbcL_mfa_gb_codon2, psbA_mfa_gb_codon1, psbA_mfa_gb_codon2
28 | GTR+I+G | 437 | 783c692a1affd190923d3956a43517a8 | psbA_mfa_gb_codon3, psbE_mfa_gb_codon3

Nexus formatted character sets for IQtree

```
#nexus
begin sets;
	charset Subset1 = 19034-19374\3 14662-15192\3 50095-50199\3 19640-19791\3 16504-16602\3 58423-59085\3 15635-16026\3 31699-31929\3 23863-24384\3 37495-37791\3 9316-9990\3 23458-23862\3 13114-13761\3 18409-18720\3 15634-16026\3 5134-5943\3 16603-16968\3 14107-14661\3 1-1911\3 59086-62193\3 18410-18720\3;
	charset Subset2 = 19376-19638\3 58424-59085\3 14108-14661\3 23459-23862\3 56714-56808\3 50096-50199\3 9317-9990\3 23864-24384\3 59087-62193\3 41543-42027\3 35681-36036\3 17915-18129\3 2-1911\3 55783-55998\3 14663-15192\3;
	charset Subset3 = 14664-15192\3 56001-56109\3 28947-29796\3 41544-42027\3 20490-22002\3 33264-34224\3 39840-40368\3 17520-17913\3 16028-16392\3 16605-16968\3 42501-43068\3 19377-19638\3 18411-18720\3 18132-18408\3 59088-62193\3 3-1911\3 18722-19032\3 36039-37494\3 17268-17517\3 20076-20487\3 50320-50409\3 19965-20073\3 19035-19374\3 15636-16026\3 13116-13761\3 5136-5943\3 26224-27423\3 58425-59085\3 5946-9315\3;
	charset Subset4 = 9993-13113\3 1912-5133\3 1914-5133\3;
	charset Subset5 = 1913-5133\3 9991-13113\3 9992-13113\3 31930-33261\3;
	charset Subset6 = 42500-43068\3 5135-5943\3 50200-50319\3 24628-25365\3 29797-31113\3 42028-42498\3 27424-28944\3 31116-31698\3 41542-42027\3 42499-43068\3;
	charset Subset7 = 5944-9315\3;
	charset Subset8 = 39839-40368\3 45218-45306\3 35680-36036\3 37792-39837\3 50322-50409\3 39838-40368\3 26225-27423\3 16969-17265\3 18723-19032\3 15195-15633\3 5945-9315\3 19639-19791\3 50321-50409\3 16505-16602\3 16971-17265\3 25366-26223\3 29799-31113\3 19792-19962\3 17267-17517\3 56385-56496\3 55665-55782\3 16506-16602\3 31700-31929\3;
	charset Subset9 = 22005-23457\3 19793-19962\3 9318-9990\3 40371-41541\3 14109-14661\3 23460-23862\3 23865-24384\3 37497-37791\3 43071-44010\3;
	charset Subset10 = 13115-13761\3 16970-17265\3 19794-19962\3 17914-18129\3 19963-20073\3 15193-15633\3 18130-18408\3 17518-17913\3 16395-16503\3;
	charset Subset11 = 16394-16503\3 17519-17913\3 56110-56232\3 56809-56994\3 28945-29796\3 13762-14106\3 16029-16392\3 56497-56586\3 43069-44010\3 40369-41541\3 16027-16392\3 18131-18408\3 15194-15633\3;
	charset Subset12 = 13763-14106\3 20489-22002\3 56384-56496\3 56713-56808\3 22004-23457\3 55411-55662\3 55412-55662\3 49850-50094\3 45398-47646\3 47648-49848\3;
	charset Subset13 = 13764-14106\3 54405-55410\3 49851-50094\3 45309-45396\3;
```

charset Subset14 = 56499-56586\3 26226-27423\3 25367-26223\3 35682-36036\3 45120-45216\3 16393-16503\3 31701-31929\3 18721-19032\3 31931-33261\3 17266-17517\3 56715-56808\3 29798-31113\3 20074-20487\3;
charset Subset15 = 43070-44010\3 42029-42498\3 16604-16968\3 27425-28944\3 56000-56109\3 19964-20073\3 40370-41541\3 28946-29796\3;
charset Subset16 = 20075-20487\3 37794-39837\3 31115-31698\3 25368-26223\3 55785-55998\3 17916-18129\3 50097-50199\3 50202-50319\3 34225-35679\3 56235-56382\3;
charset Subset17 = 19375-19638\3 19033-19374\3;
charset Subset18 = 19641-19791\3;
charset Subset19 = 45397-47646\3 52987-54402\3 56587-56712\3 56995-58422\3 20488-22002\3 22003-23457\3 47647-49848\3 51469-52986\3;
charset Subset20 = 54403-55410\3 55999-56109\3 55663-55782\3 44011-44646\3 24385-24627\3 44647-45117\3 49849-50094\3;
charset Subset21 = 44648-45117\3 56383-56496\3 56588-56712\3 44012-44646\3 56111-56232\3 24386-24627\3 55664-55782\3 54404-55410\3 52988-54402\3 51470-52986\3;
charset Subset22 = 52989-54402\3 45399-47646\3 27426-28944\3 24630-25365\3 44649-45117\3 56997-58422\3 56811-56994\3 24387-24627\3 56112-56232\3 44013-44646\3 56589-56712\3;
charset Subset23 = 45119-45216\3 45118-45216\3 45307-45396\3 45308-45396\3 56810-56994\3 55784-55998\3 56234-56382\3 50201-50319\3 56498-56586\3 36038-37494\3 37496-37791\3 34227-35679\3 31114-31698\3 24629-25365\3 33263-34224\3;
charset Subset24 = 31932-33261\3;
charset Subset25 = 37793-39837\3 33262-34224\3 56233-56382\3 45217-45306\3 34226-35679\3 36037-37494\3;
charset Subset26 = 45219-45306\3 51471-52986\3 47649-49848\3 42030-42498\3;
charset Subset27 = 56996-58422\3 50410-51468\3 50411-51468\3;
charset Subset28 = 50412-51468\3 55413-55662\3;
charpartition PartitionFinder = GTR+I+G:Subset1, GTR+I+G:Subset2, GTR+I+G:Subset3, GTR+I+G:Subset4, GTR+I+G:Subset5, GTR+I+G:Subset6, GTR+I+G:Subset7, GTR+I+G:Subset8, GTR+I+G:Subset9, GTR+I+G:Subset10, GTR+G:Subset11, GTR+I+G:Subset12, GTR+I+G:Subset13, GTR+I+G:Subset14, GTR+G:Subset15, GTR+I+G:Subset16, GTR+G:Subset17, GTR+I+G:Subset18, GTR+I+G:Subset19, GTR+I+G:Subset20, GTR+G:Subset21, GTR+I+G:Subset22, GTR+G:Subset23, GTR+I+G:Subset24, GTR+I+G:Subset25, GTR+I+G:Subset26, GTR+I+G:Subset27, GTR+I+G:Subset28;
end;
