# Supplementary figures and images for: Plastid Phylogenomics and Plastomic Diversity of the Extant Lycophytes
Source: Genes (Basel). 2022 Jul 19;13(7):1280. doi: 10.3390/genes13071280 (PMC9316050; doi:10.3390/genes13071280)

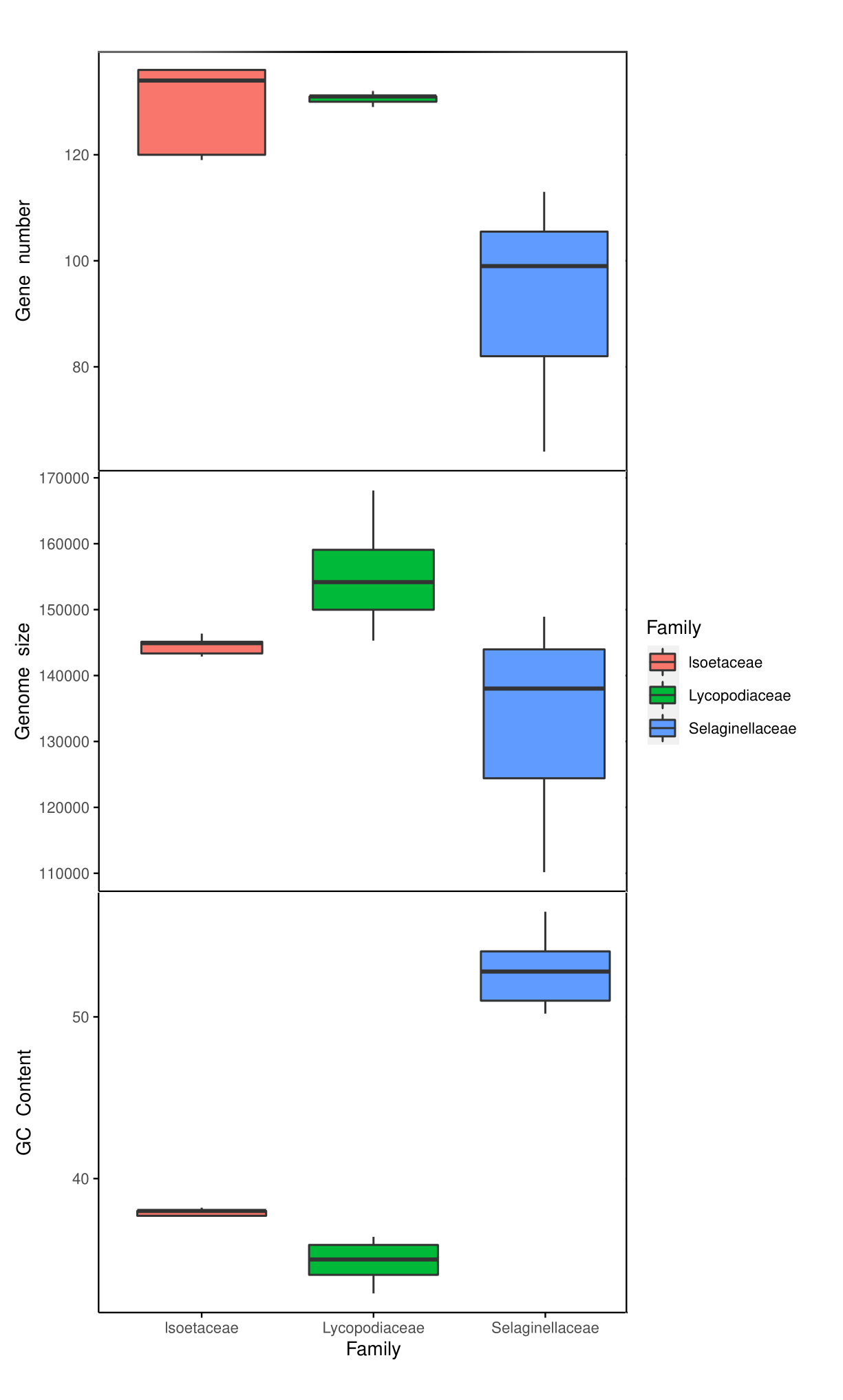

Supplement: Supplementary file 1 [file genes-13-01280-s001.zip › Figure S1.jpg]

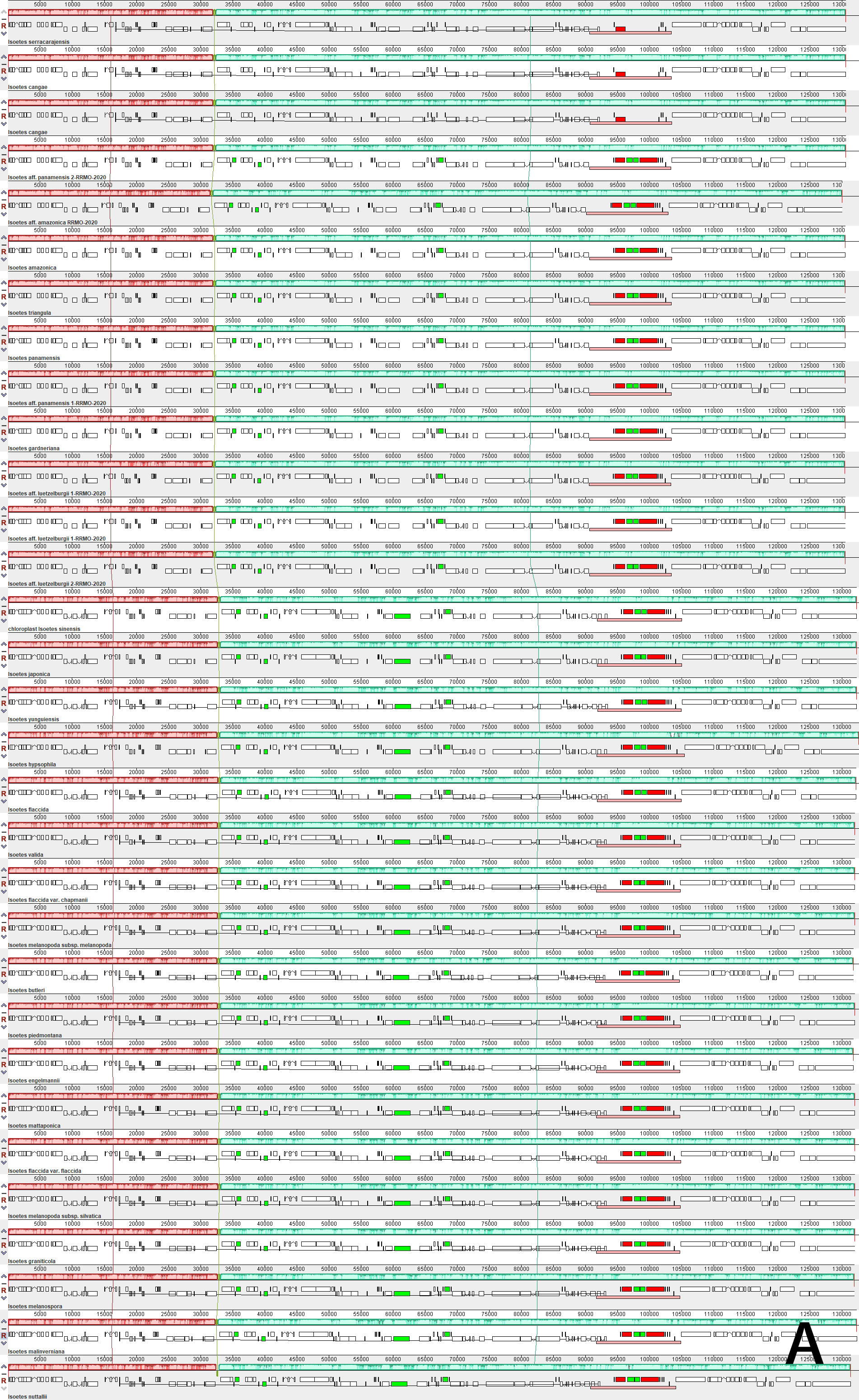

Supplement: Supplementary file 1 [file genes-13-01280-s001.zip › Figure S2A.tif]

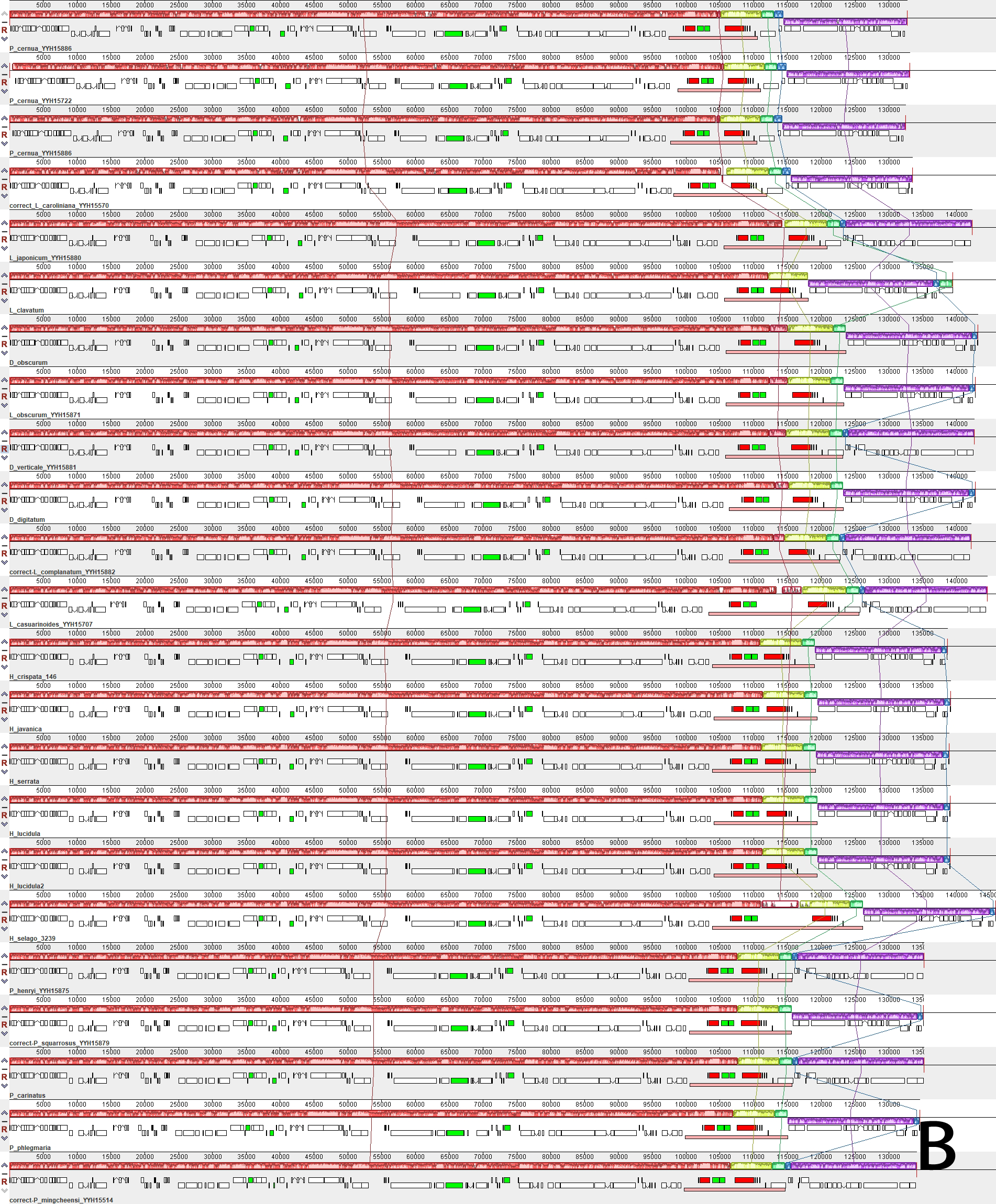

Supplement: Supplementary file 1 [file genes-13-01280-s001.zip › Figure S2B.tif]

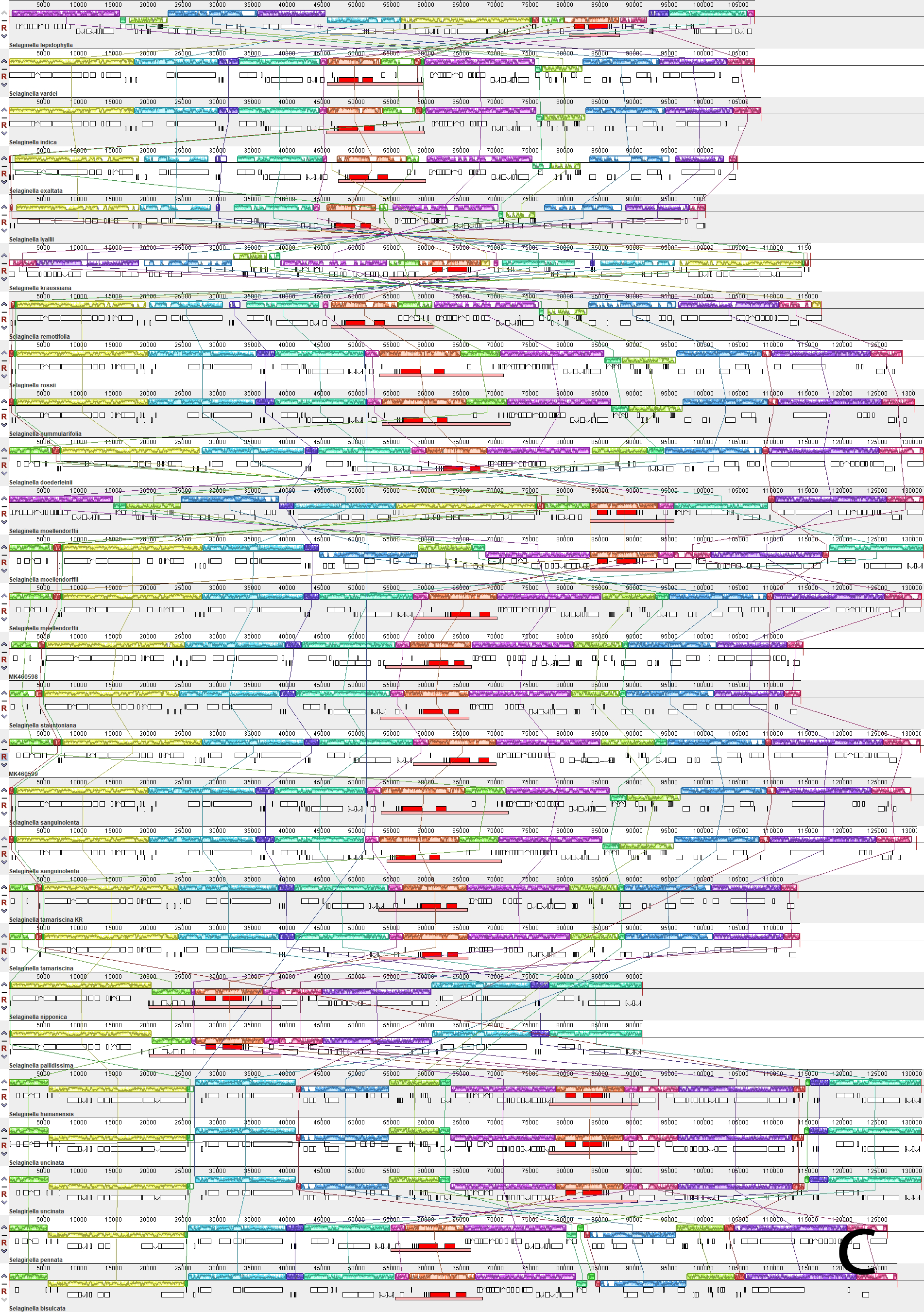

Supplement: Supplementary file 1 [file genes-13-01280-s001.zip › Figure S2C.tif]
